# Mechanism of Notch Pathway Activation and Its Role in the Regulation of Olfactory Plasticity in *Drosophila melanogaster*

**DOI:** 10.1371/journal.pone.0151279

**Published:** 2016-03-17

**Authors:** Simon Kidd, Toby Lieber

**Affiliations:** Department of Genetics and Development, Columbia University College of Physicians and Surgeons, 701 West 168th Street, New York, New York, United States of America; Center for Genomic Regulation, SPAIN

## Abstract

The neural plasticity of sensory systems is being increasingly recognized as playing a role in learning and memory. We have previously shown that Notch, part of an evolutionarily conserved intercellular signaling pathway, is required in adult *Drosophila melanogaster* olfactory receptor neurons (ORNs) for the structural and functional plasticity of olfactory glomeruli that is induced by chronic odor exposure. In this paper we address how long-term exposure to odor activates Notch and how Notch in conjunction with chronic odor mediates olfactory plasticity. We show that upon chronic odor exposure a non-canonical Notch pathway mediates an increase in the volume of glomeruli by a mechanism that is autonomous to ORNs. In addition to activating a pathway that is autonomous to ORNs, chronic odor exposure also activates the Notch ligand Delta in second order projection neurons (PNs), but this does not appear to require acetylcholine receptor activation in PNs. Delta on PNs then feeds back to activate canonical Notch signaling in ORNs, which restricts the extent of the odor induced increase in glomerular volume. Surprisingly, even though the pathway that mediates the increase in glomerular volume is autonomous to ORNs, nonproductive transsynaptic Delta/Notch interactions that do not activate the canonical pathway can block the increase in volume. In conjunction with chronic odor, the canonical Notch pathway also enhances cholinergic activation of PNs. We present evidence suggesting that this is due to increased acetylcholine release from ORNs. In regulating physiological plasticity, Notch functions solely by the canonical pathway, suggesting that there is no direct connection between morphological and physiological plasticity.

## Introduction

Odors are key environmental signals that influence animal behavior. Attractive odors signal the presence of food or a potential mate. Aversive odors warn of predators or toxins (reviewed in [1]). Repeated exposure to odors in the absence of an associated reward or punishment leads to reduced behavioral response to that odor (reviewed in [2]). Animals can also learn to associate odors with aversive or appetitive stimuli (reviewed in [3]). Depending on odor valence and the exposure paradigm, chronically exposing an animal to odor will lead to either reduced behavioral responsiveness or increased behavioral attraction to subsequent presentations of that odor [4–10]. While the role of plasticity at central brain synapses in mediating olfactory learning and memory has been well established ([11,12], reviewed in [13,14]), recent work has highlighted the importance of plasticity at the first olfactory synapse that provides primary olfactory input to the brain [10,15–18].

**Fig 1:**
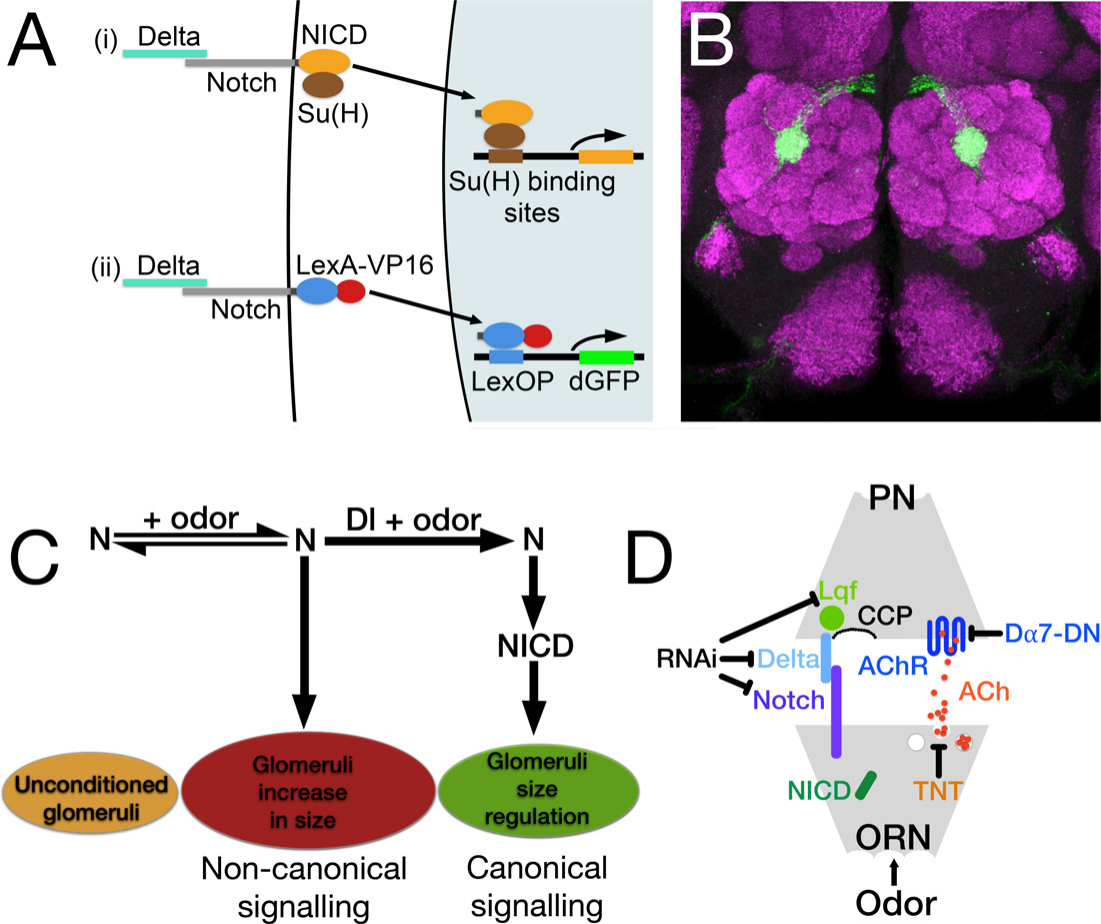
Notch reporter assay, the Notch pathway in the regulation of glomeruli size and the experiments described in this paper. (**A**) Diagram of the canonical Notch pathway and reporter assay. (i) Proteolytic cleavages of Notch (N), upon binding its ligand Delta (Dl), result in the cytoplasmic domain of N (NICD), along with its transcriptional effector Suppressor of Hairless (Su(H)), entering the nucleus and activating transcription of genes with Su(H) binding sites. (ii) In the Notch reporter protein, NICD is replaced with LexA-VP16. The reporter is under control of the α-tubulin promoter and is expressed at low levels in most if not all cells. Like N, the reporter protein binds endogenous Dl, and the consequential cleavage releases LexA-VP16, which enters the nucleus and activates expression of destabilized GFP (dGFP) from a LexOP.dGFP reporter. (**B**) The central brain of a fly carrying the N reporter protein after 4 days of exposure to geranyl acetate. N reporter activity can be seen in the axons of olfactory receptor neurons (ORNs) that project to glomerulus VA6. The antennal lobe has been stained with anti-Bruchpilot monoclonal antibody (nc82; magenta). This labels presynaptic active zones [81]. N reporter activity was detected with anti-GFP antibody (green). (**C**) This diagram illustrates the interplay between the non-canonical and canonical N pathways in the regulation of the chronic odor induced increase in glomerular volume (see text). (**D**) A cartoon of a synapse depicting the locations of the proteins manipulated in this study. N is presynaptic while Dl and Liquid Facets (Lqf), the fly epsin homologue, are postsynaptic. All three were knocked down by RNAi. The acetylcholine receptor, nAChR, is postsynaptic and its function was knocked down by a dominant negative form of the Dα7 subunit (Dα7-DN). To show the effect of constitutively activating N, its cytoplasmic domain, NICD, was expressed presynaptically in the ORN. Tetanus toxin light chain (TNT) was expressed presynaptically to block vesicle release of acetylcholine (ACh) and neuropeptides. PNs, Projection Neurons; ORNs, Olfactory Receptor Neurons; CCP clathrin coated pits which are necessary for the Lqf dependent endocytosis of Dl [82]. Protein colors match those used in subsequent figures.

Flies detect odors via olfactory receptor neurons (ORNs) that decorate the antenna and maxillary palps. These can be divided into approximately 50 subclasses based on the particular olfactory receptor (OR) they express. All the axons of ORNs expressing the same OR project to the same glomerulus, a morphologically distinct neuropil region of the antennal lobe (Fig 1B). Here they synapse with the dendrites of second order projection neurons (PNs) that relay information to the mushroom body and lateral horn and with the dendrites of local interneurons (LNs) that innervate many glomeruli and mediate cross talk between them ([19–21], reviewed in [22]). The exposure of animals to odors, using paradigms that result in either habituation [4,6,23], or associative conditioning [10,17,24–26] also leads to odor specific increases in glomerular volume. In addition, exposure to odors using paradigms that result in habituation lead to odor specific decreases in physiological responses of PNs [23], while exposure to odors using an associative conditioning paradigm leads to odor specific increases in PN activation [10].

We previously showed that Notch is required in *Drosophila* ORNs for changes in glomerular volume that occur as a consequence of chronic exposure to concentrations of CO_2_ that are aversive or to concentrations of geranyl acetate that are attractive. Using an associative conditioning paradigm in which flies associate odor with food, and that consequently results in enhanced behavioral attraction to geranyl acetate, we further showed that flies that had been chronically exposed to geranyl acetate exhibited an increase in PN activation that is dependent on Notch in ORNs [10]. Notch is a transmembrane protein that plays an essential role during development and is been increasingly recognized as playing a role in synaptic plasticity and memory [10,27–42]. In the canonical Notch pathway, Notch is activated by binding to the transmembrane ligands Delta or Serrate that are presented on neighboring cells. Notch then undergoes a series of proteolytic cleavages that result in the cytoplasmic domain of Notch entering the nucleus in association with the transcriptional effector Suppressor of Hairless (Su(H)) and promoting the transcription of genes with Su(H) binding sites (Fig 1A(i)) (reviewed in [43]). There are also non-canonical modes of Notch function that do not utilize all the components of the canonical Notch pathway and involve interactions with other molecules [44–50].

In regulating the odor induced increase in glomerular volume, we previously demonstrated that Notch functions by both non-canonical (Delta and cleavage independent) and canonical (Delta and cleavage dependent) mechanisms. Non-canonical Notch signaling is required for the increase in volume, and canonical Notch signaling regulates the extent of the increase (Fig 1C). The canonical Notch ligand Delta that is expressed in PNs switches the balance of Notch activity in ORNs from the non-canonical to the canonical pathway (Fig 1C and 1D). Using calcium imaging, we further showed that Notch in ORNs is required for an increase in PN activation that occurs as a consequence of chronic odor induced changes in PN activation. Our data defined a circuit whereby, in conjunction with odor, Notch activity in the periphery regulates the activity of neurons in the central brain and Delta in the central brain feeds back to regulate N activity in the periphery [10].

Having established that Notch in ORNs responds to environmental inputs, i.e. odors, we set out here to determine how this happens. How does exposure to odor activate non-canonical and canonical Notch signaling? We present data indicating that the odor induced increase in volume that is mediated by non-canonical Notch signaling does not appear to require ORN output, suggesting that it is autonomous to the ORN. Despite this autonomy, nonproductive transsynaptic Delta/Notch interactions can block the increase in volume that is mediated by the non-canonical pathway. In contrast to what we observed for the non-canonical pathway, activation of the canonical pathway, and the resulting restriction of the extent of the odor induced increase in glomerular volume, does require ORN output. However, rather than Delta being activated in response to cholinergic activation of PNs by their cognate ORNs, our data lead us to hypothesize that Delta is activated by a neuropeptide released from the ORNs. We also present evidence for the existence of a pathway in PNs that acts in conjunction with the canonical Notch pathway in ORNs to regulate the extent of the odor induced increase in glomerular volume.

We then asked if in regulating physiological plasticity Notch, as it does in regulating morphological plasticity, also functions by both canonical and non-canonical mechanisms. Our data show that in mediating the increase in PN activation that occurs as a consequence of prior long-term odor exposure Notch acts solely via the canonical pathway, suggesting that there is no direct connection between morphological and physiological plasticity. We further present data suggesting that the canonical Notch pathway regulates the level of acetylcholine released by ORNs. We propose that odor induced release of a neuropeptide from ORNs activates Delta in PNs. Delta then activates the canonical Notch pathway in ORNs, which increases the amount of acetylcholine released. The Notch induced increase in acetylcholine release acts in conjunction with odor induced increase in acetylcholine release to enhance PN activation.

## Results

### PN activation is required for the regulation of glomerular volume

In regulating the long-term odor induced increase in volume of glomeruli, the role of the canonical Notch pathway is to restrict the extent of the increase. Knocking down Delta in PNs results in a greater odor induced increase in glomerular volume than is observed in control flies (Fig 1C) [10]. We asked how long-term odor exposure activates Delta in PNs to regulate glomerular volume. Expression of tetanus toxin light chain in adult ORNs blocked activation of a Notch reporter that monitors canonical, Delta dependent Notch pathway activation (Fig 1A) [51]. Because tetanus toxin prevents synaptic vesicle release (Fig 1D), we hypothesized that Delta activation in PNs and canonical Notch pathway regulation of glomerular volume is induced by activation of the PNs by odor mediated synaptic transmission from ORNs [10].

**Fig 2:**
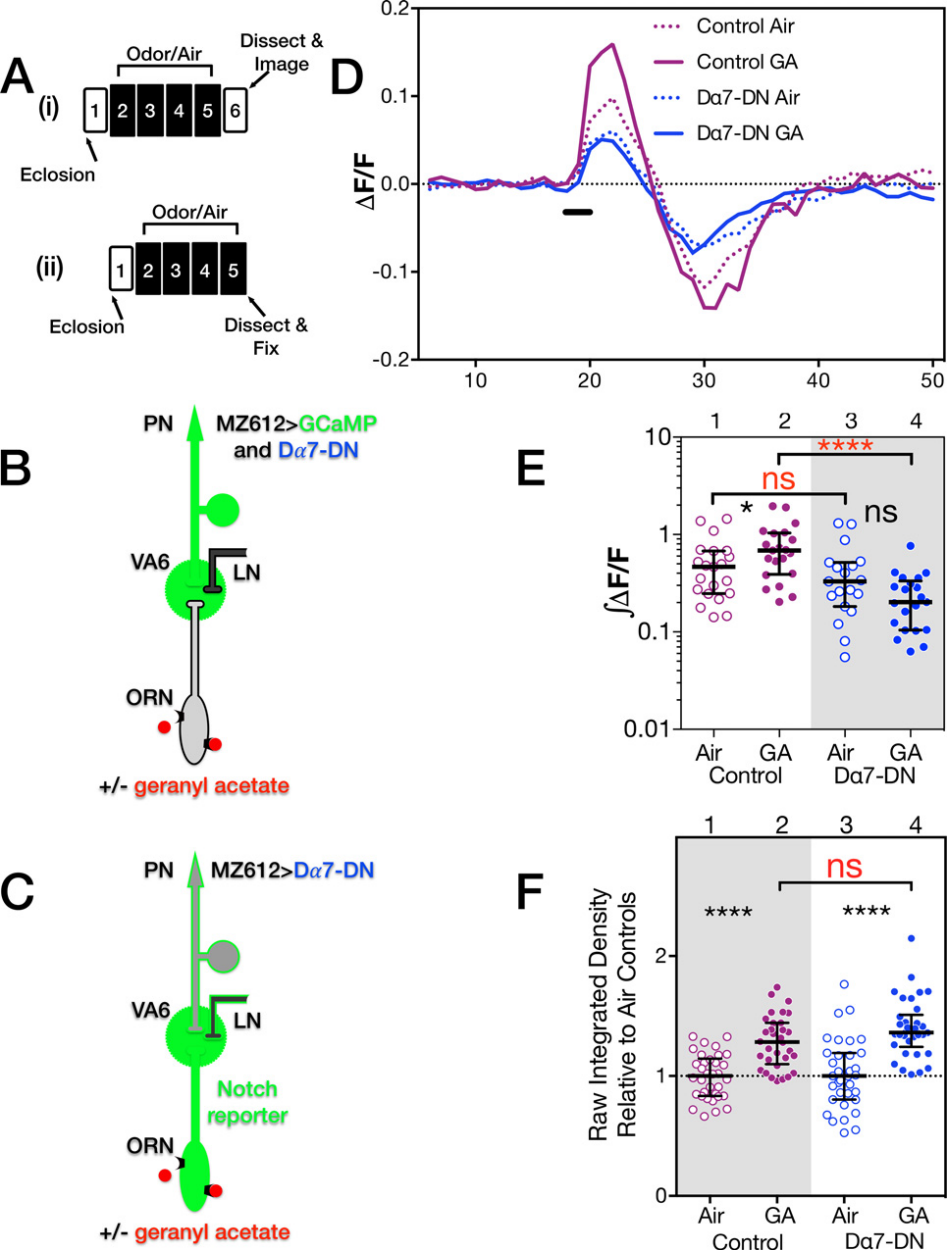
Expression in PNs of a dominant negative Dα7 subunit of nAChR blocks Ca^2+^ influx into PNs but not Notch reporter activity in ORNs. (**A**) Schematics of the experimental protocols used for (i) calcium imaging and (ii) the N reporter assay. Each day is represented by a numbered block; shaded blocks are those where odor was applied. (**B** and **C**) The relevant genotypes are shown on cartoons of the olfactory circuit. In (**D** and **E**) female flies were exposed to 1% geranyl acetate (GA) in paraffin oil or paraffin oil alone and removed from odor for one day prior to imaging. Flies (as illustrated in **B**) were MZ612-GAL4 UAS.GCaMP6s/Or82a-LexAGAD; UAS.GCaMP6s LexOP.dsRED without (control) or with UAS.Dα7-DN (Dα7-DN). The dsRED reporter was used to identify VA6. For the calcium imaging experiments and for the reporter assays below, we assayed approximately 20 glomeruli for each combination of odor exposure and genotype. The traces (**D**) depict the median ΔF/F for each genotype and condition over time. (The numbers on the x-axis are frames of 472 msec each). The black bar indicates the time of a 1.5 second GA pulse; solid traces, GA exposed flies; dotted traces, air exposed flies; magenta traces, control; blue traces, Dα7-DN. (**E**) ∫ΔF/F, plotted in log scale, of calcium influx and efflux. Here and in the figures below the data are displayed as scatter plots with median and interquartile ranges and were compared by Mann-Whitney tests. Each pair of lanes represents air exposed flies (open circles) and the corresponding GA exposed flies (filled circles). p-values were determined for GA exposed versus air exposed flies by comparing the areas under the influx peaks for each glomerulus. Here and in the figures below, ns p>0.05; * p 0.05; ** p 0.01; *** p 0.001; **** p 0.0001. The statistics were based on the following number of glomeruli; lane 1, 21; lane 2, 19; lane 3, 19; lane 4, 21. In (**F**) one day old female flies were exposed to 1% GA in paraffin oil or paraffin oil alone for 4 days and Notch reporter activity determined. Flies (as illustrated in **C**) were MZ612-GAL4; N-LV LexOP.dGFP carrying either UAS.Val20 (control) or UAS.Dα7-DN (Dα7-DN). The plot of each air exposed/odor exposed pair has been normalized to the median of the air exposed flies. Their median at 1 is indicated by the dashed line. This normalization allows us to compare the volumes of the odor exposed flies in each experiment.

Since ORN to PN synapses are cholinergic [52], we addressed the requirement for PN activation by using a dominant negative form of the Dα7 subunit of the nicotinic acetylcholine receptor (nAChR; Y195T [53]; Dα7-DN, Fig 1D). Flies that have been chronically exposed to geranyl acetate exhibit an increase in the amplitude of calcium activity in PNs in response to subsequent presentation of geranyl acetate compared to air exposed controls [10]. To confirm that expression of Dα7-DN in PNs affected activation of PNs by ORNs following long-term odor exposure, we used the GAL4 system to express Dα7-DN along with GCaMp6s [54] in the PNs that innervate VA6 (VA6 PNs; Fig 2B). We exposed flies to air or geranyl acetate for 4 days, allowed them to recover in the absence of odor for one day, and then used 2-photon microscopy to measure calcium responses in VA6 PN dendrites following transient presentation of geranyl acetate (Fig 2A(i)).

In air exposed flies, upon presentation of geranyl acetate, there was no statistically significant difference in the amplitude of calcium activity in PNs between control flies and those expressing Dα7-DN. This can be seen in the traces of the median fluorescent change over time (ΔF/F; compare dashed blue trace, Dα7-DN, with dashed purple trace, control, in Fig 2D) and the plots of ∫ΔF/F (Fig 2E, lanes 1 and 3).

We then asked what effect the expression of Dα7-DN had on the activation of PNs in flies that had been chronically exposed to odor. Control flies chronically exposed to geranyl exhibit an increase in the amplitude of calcium activity in PNs in response to subsequent presentation of geranyl acetate compared to air exposed controls (Fig 2D and 2E; compare dashed purple trace, air exposed controls, with solid purple trace, geranyl acetate exposed controls, in Fig 2D, and lanes 1 and 2 in the plots of ∫ΔF/F in Fig 2E and Kidd et al. 2015 [10]). Exposing flies on food to geranyl acetate for 4 days results in enhanced behavioral attraction to geranyl acetate [10] indicating that the flies associate geranyl acetate with food. This might be reflected in enhanced activation of VA6 PNs.

Unlike control flies, in flies expressing Dα7-DN there was no difference in PN activation between flies exposed to geranyl acetate or air. This is depicted in the traces of ΔF/F (Fig 2D), where the solid blue trace (geranyl acetate exposed flies) overlaps with the dashed blue trace (air exposed flies), and in the plots of ∫ΔF/F (Fig 2E, lanes 3 and 4). Thus, the expression of Dα7-DN in VA6 PNs affects the activation of those PNs in flies that had been chronically exposed to geranyl acetate. We attribute the observation that the expression of Dα7-DN did not affect the activation of PNs in air exposed flies to there being sufficient wild type nAChR to bind to the acetylcholine (ACh) released. This is no longer the case when higher levels of ACh are released upon chronic exposure to geranyl acetate.

**Fig 3:**
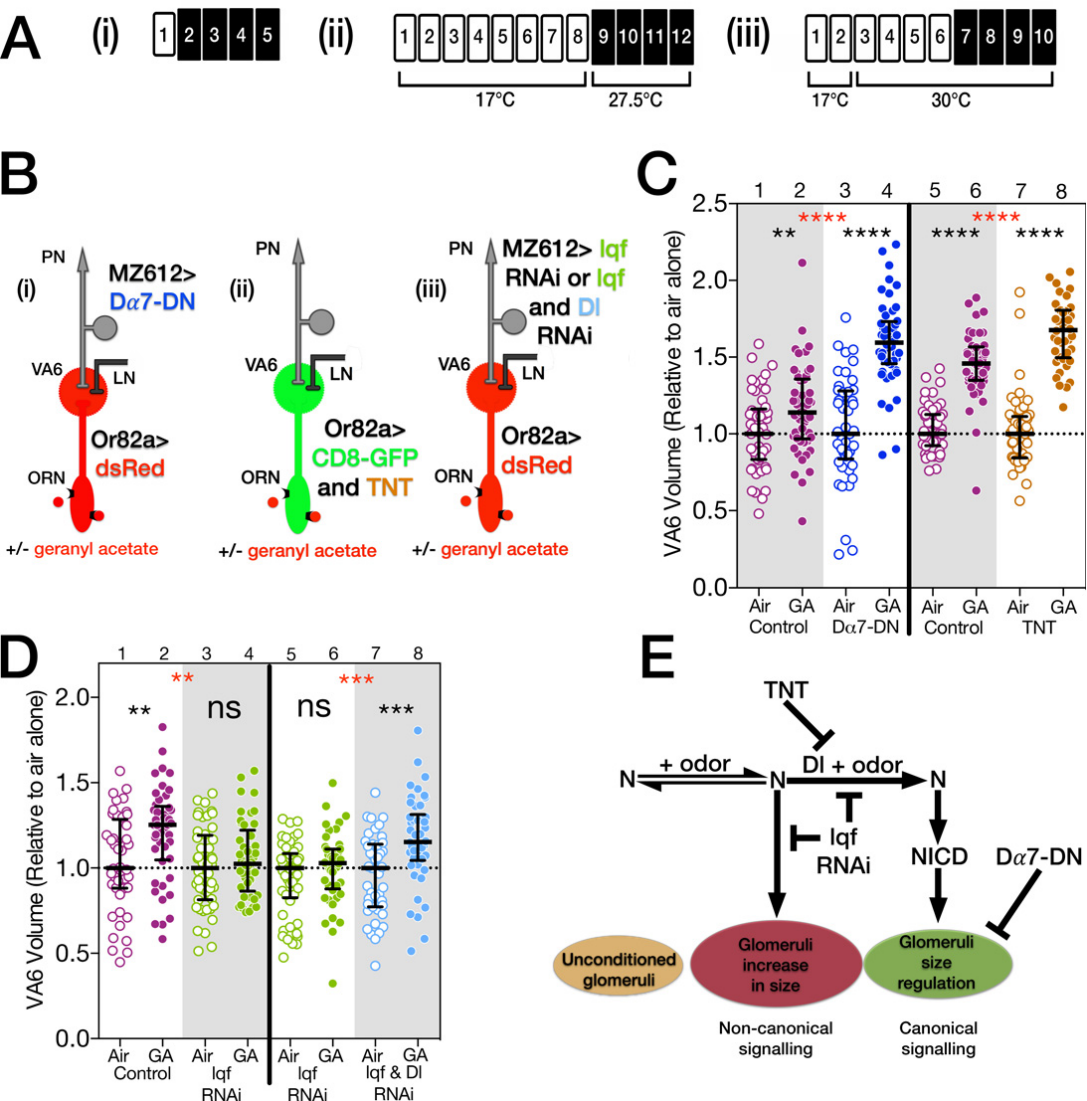
Lqf/epsin in PNs is required for the odor induced increase in glomeruli volume, while Ach release from ORNs and nAchR activation in PNs are required to restrict the extent of the increase. (**A**) Schematics of experimental protocols. (**B**) Cartoons of the olfactory circuit depicting the relevant genotypes. (**C** and **D**) Scatterplots of VA6 volumes. The data were normalized as described in Fig 2. Magenta circles, controls; blue circles, Dα7-DN; brown circles, tetanus toxin; green circles, *lqf* RNAi; pale blue circles, both *lqf* and *Dl* RNAi. Empty circles, air exposed; filled circles, GA exposed. Black p values compare air and GA exposed samples of the same genotype. Red p values compare GA exposed samples between genotypes. In **C, lanes 1–4**, one day old female flies were exposed to 1% GA in paraffin oil or paraffin oil alone and the volumes of VA6 determined. Flies, as illustrated in **B(i)**, were MZ612-GAL4; Or82a-LexAGAD LexOP.dsRED carrying either UAS.Val20 (control) or UAS.Dα7-DN (Dα7-DN). In **C, lanes 5–8**, flies which had been grown and then maintained at 17°C for eight days were exposed to air or GA at 27.5°C. Flies, as illustrated in **B(ii)**, were Or82a-GAL4/tubP-gal80^ts20^; UAS.mCD8-GFP with UAS.IMP (inactive tetanus toxin, control) or UAS.TNT (active tetanus toxin, TNT). The temporal protocol was chosen, because we found empirically that younger flies were more susceptible to axonal damage induced by the expression of tetanus toxin. The lower temperature used to inactivate the GAL80^ts^ reduced the amount of tetanus toxin expressed and and reduced the amount of axonal damage. In **D,** flies that had been grown and then maintained at 17°C for 2 days, were shifted to 30°C for 4 days prior to being exposed to 1% GA or air for a further 4 days. Flies, as illustrated in **B(iii)**, were MZ612-GAL4/tubP-gal80^ts10^; Or82a-LexAGAD LexOP.dsRED/UAS.dicer-2 with both UAS.epsin RNAi and UAS.Dl shRNA (*lqf* & *Dl* RNAi, lanes 7 and 8), just UAS.epsin RNAi (*lqf* RNAi, lanes 3–6), or neither (control, lanes 1 and 2). (**E**) Diagram of the non-canonical and canonical N pathways together with the results described in this figure and their relationship to odor induced glomeruli volume change.

Having established that expression of Dα7-DN in VA6 PNs affected the activation of those PNs in flies that had been chronically exposed to geranyl acetate, we asked what affect expression of Dα7-DN in VA6 PNs had on the increase in glomerular volume that is induced by long-term odor exposure. In other words, is the enhanced activation of VA6 PNs that occurs in geranyl acetate control flies, but not in Dα7-DN expressing flies, required to restrict the odor induced increase in glomerular volume? We used a VA6 PN GAL4 driver to express Dα7-DN in VA6 PNs and measured the volume of VA6 by using Or82a-LexA-GAD to drive expression of dsRED in VA6 ORNs (Fig 3B(i)). Control flies or flies expressing Dα7-DN were exposed to air or geranyl acetate for 4 days (Fig 3A(i)). VA6 volumes are plotted in Fig 3C lanes 1–4. (Here and in figures below, the volumes of all the glomeruli were normalized to the median volume of the glomeruli of unexposed flies. The normalized median volume of the glomeruli of the unexposed flies is by definition 1 and is indicated by the dashed line). The increase in volume of VA6 was larger in flies expressing Dα7-DN than in control flies (lanes 2 and 4). This indicates that the enhanced cholinergic activation of VA6 PNs that occurs as a consequence of chronic odor exposure is required to restrict the extent of the increase in glomerular volume. The phenotype of flies expressing Dα7-DN in VA6 PNs is similar to that of flies expressing *Delta* RNAi in VA6 PNs [10], suggesting that cholinergic activation of PNs activates Delta.

### PN activation is not required for Delta activation

To directly test whether activation of PNs activates Delta, we asked whether expression of Dα7-DN in VA6 PNs affected activation of the Notch reporter in flies that had been chronically exposed to geranyl acetate. The reporter is a direct readout of Delta activation. We used GAL4 to drive expression of Dα7-DN in VA6 PNs and assayed Notch reporter activity using our Notch-LexA-VP16 reporter assay (Figs 1A(ii) and 2C). As can be seen in Fig 2F, in flies expressing Dα7-DN exposure to geranyl acetate still resulted in activation of the Notch reporter. Thus, despite the fact that expression of tetanus toxin in ORNs blocks Delta activation, the continued reporter activity indicates that enhanced cholinergic activation of VA6 PNs is not required. While our previous results showed that the principal source of Delta that activates the Notch reporter is PNs, and not LNs, ORNs, or glia [10], we cannot exclude the possibility that in the absence of increased PN activation, the Notch reporter is being activated by Delta from another cell type. However, we favor the hypothesis that upon chronic odor exposure, rather than being activated by neurotransmitter mediated PN activation, Delta in PNs is activated by a neuropeptide released by ORNs. Tetanus toxin, as well as affecting neurotransmitter release, has also been shown to affect the release of neuropeptides [55,56].

Dα7-DN and *Delta* RNAi expressing flies both have the same phenotype with respect to the chronic odor induced increase in glomerular volume. Both result in volumes that are larger than that of control flies. However, the expression of Dα7-DN does not block Delta activation. This indicates that enhanced ACh mediated PN activation is not upstream of Delta activation. The data suggest that either the canonical Notch pathway and ACh mediated PN activation regulate glomerular volume by parallel pathways, or the canonical Notch pathway is upstream of cholinergic PN activation, or a combination of the two. We present data below that suggests that in addition to its independent role in regulating glomerular volume, Delta activation is upstream of enhanced PN activation.

### The increase in volume of glomeruli that is induced by long-term odor exposure does not require ORN activation of downstream neurons

We have previously shown that a non-canonical Notch pathway in ORNs is required for the increase in the volume of glomeruli that occurs as a consequence of chronic odor exposure (Fig 1C) [10]. The fact that VA6 still increases in volume in odor exposed flies expressing Dα7-DN in VA6 PNs indicates that cholinergic activation of PNs is not required for the increase in volume, and suggests that the non-canonical Notch pathway does not require PN activation. This raises the possibility that activation of neurons downstream of the ORNs is not required for the odor induced activation of the non-canonical Notch pathway that mediates the increase in glomerular volume. To test this hypothesis we used GAL80^ts^ and Or82a-GAL4 to express either active (TNT) or inactive (IMP) forms of the tetanus toxin light chain in adult VA6 ORNs and measured the volume of VA6 following exposure to air or geranyl acetate by co-expressing CD8-GFP in the ORNs (Fig 3A(ii) and 3B(ii)). In flies exposed to geranyl acetate the volume of VA6 still increased even when synaptic vesicle release was blocked by expression of TNT (Fig 3C, lanes 7 and 8), indicating that activation of downstream neurons is not required for the volume increase or for activation of the non-canonical Notch pathway. If there is a requirement for a ligand to activate the non-canonical pathway, activation of the ligand does not require activation of neurons downstream of ORNs. These observations raise the possibility that the activation of the non-canonical Notch pathway and the concomitant odor induced increase in glomerular volume are autonomous to ORNs. Expression of TNT blocks both Delta activation [51] and ACh mediated PN activation, and therefore as predicted the increase in volume of VA6 in flies expressing TNT is greater than that of control flies (Fig 3C, lanes 6 and 8).

### Nonproductive transsynaptic Delta/Notch interactions block the chronic odor induced increase in glomerular volume

Odor dependent activation of the canonical Notch pathway in VA6 ORNs, as assayed by activation of the Notch reporter, is dependent on Epsin in VA6 PNs [10]. Epsin is an endocytic protein that mediates Delta internalization through a specific endocytic route and is required for Delta activation of the canonical Notch pathway (Fig 1D) [57,58]. The prediction was therefore, that RNAi mediated knock down of Epsin (encoded in flies by *liquid facets*, *lqf*) in VA6 PNs should have the same phenotype as knocking down Delta in those PNs, i.e. that upon chronic exposure to geranyl acetate the volume of VA6 glomeruli would be larger than that of control flies. Surprisingly, however, when we used RNAi to knock down Lqf in adult VA6 PNs, exposed flies to geranyl acetate, and measured the volume of VA6 (Fig 3A(iii) and 3B(iii)), VA6 did not increase in volume (Fig 3D, lanes 1–4); the phenotype resembled that of loss of Notch in ORNs rather than loss of Delta in PNs. It is thought that Lqf is required specifically for canonical Notch pathway signaling [57,58]. To ensure that this is indeed the case i.e. that knocking down Lqf in VA6 PNs is affecting the increase in the volume of VA6 via its interaction with Delta, rather than by an alternate mechanism, we knocked down both Delta and Lqf in adult VA6 PNs (Fig 3B(iii)). When we measured the volume of VA6 following exposure of these flies to geranyl acetate for 4 days (Fig 3A(iii)), we found that VA6 once again increased in volume (Fig 3D lanes 5–8), i.e. the phenotype resembled the loss of Delta. These data demonstrate that not only is Lqf required for canonical Notch signaling, but in its absence non-canonical signaling that is Delta independent and does not require PN activation is also blocked (Fig 3E). As we expand upon in the discussion, we hypothesize that in the presence of odor and in the absence of Lqf, Delta in PNs forms a non-productive complex across the synapse with Notch in ORNs that sequesters Notch away from components of the non-canonical Notch pathway thereby blocking the increase in glomerular volume.

### Delta is required for the reversibility of odor induced increases in glomerular volume

**Fig 4:**
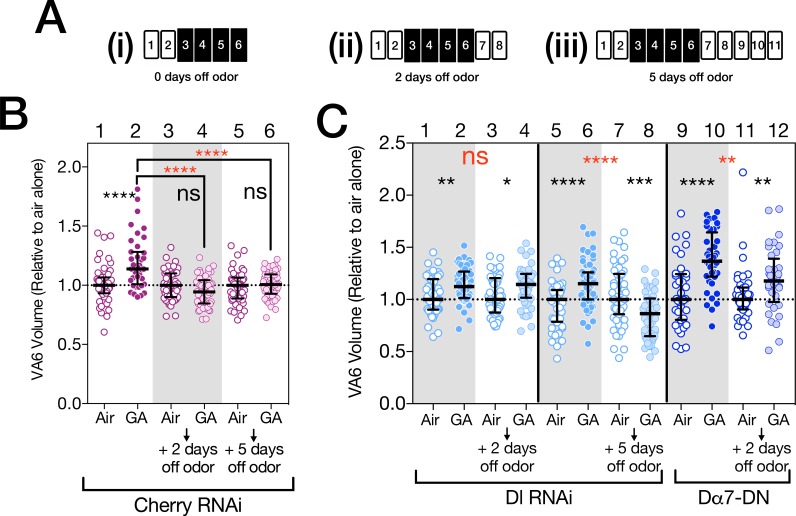
Delta and nAChRs are required in PNs for the reversibility of odor induced increases in glomerular volume. (**A**) Schematics of experimental protocols. Two day old females were exposed to 1% GA in paraffin oil or paraffin oil alone for 4 days, removed form odor for the indicated times and VA6 volumes determined. (**B** and **C**) Scatterplots of glomeruli volumes. These have been normalized as described in Fig 2. Empty circles, air exposed; filled circles, GA exposed; lightly shaded circles, flies that had been removed from odor. (**B**) Control flies (magenta; first four lanes are taken from Kidd et al., 2015 [10], but the experiment depicted in lanes 5 and 6 was done at the same time, i.e. all the flies are from the same population). (**C, lanes 1–8**) *Dl* RNAi flies (pale blue). Two separate experiments are shown. In lanes 1–4 flies were removed from odor for 2 days. In lanes 5–8 flies were removed from odor for 5 days. (**C, lanes 9–12)** Dα7-DN flies (blue). In the four experiments depicted in **B** and **C** flies in each experiment are from the same population, and for each comparison of flies, with or without odor exposure, they are the same age. Because for logistical reasons wild type flies were not included in the experiments depicted in **C,** we are not unable to determine the fold change relative to wild type flies. The control flies in **B** expressed CD8-GFP and mCherry shRNA in VA6 ORNs under control of Or82a-GAL4. The *Dl* RNAi flies in **C** were MZ612-GAL4; Or82a.CD8-GFP/UAS.Dl shRNA. The Dα7-DN flies in **C** were MZ612-GAL4; Or82a.CD8-GFP/UAS.Dα7-DN.

We and others have shown that the increase in the volume of glomeruli is reversible when flies are removed from odor for 2 days (Fig 4B, lanes 1–4) [6,10]. This reversal could be due to a gradual winding down of the pathway that promotes the volume increase. Alternatively Delta, by activating the canonical Notch pathway to restrict the volume increase, could also be actively required for the reversion in volume. To distinguish between these alternatives, we used *Delta* RNAi to knock down Delta in VA6 PNs, exposed the flies to air or geranyl acetate for 4 days, removed them from odor for 2 days, and measured the volume of VA6 by using a direct fusion of CD8-GFP to the Or82a promoter (Fig 4A(ii)) [59]. As shown in Fig 4C, lanes 1–4, when Delta is knocked down in VA6 PNs, the volume of VA6 does not decrease when flies are removed from odor for 2 days. This indicates that for at least up to 2 days there is a requirement for Delta for the reversion in glomeruli volume. We have suggested above, that Delta in PNs, rather than being activated by synaptic transmission form ORNs to PNs, is activated by neuropeptide release by ORNs. Neuromodulators can act on a longer time scale than do neurotransmitters, which could account for Delta still being active when flies are removed from odor for 2 days. When flies are removed from odor for 5 days, Delta is no longer required for the decrease in volume of VA6 (Fig 4C, lanes 5–8), presumably because by that time the process promoting the volume increase has indeed wound down.

We have shown above that a pathway in PNs that is activated by cholinergic activation also restricts the extent of the odor induced increase in glomerular volume. Surprisingly, when the increase in PN activation induced by odor is blocked by the expression of Dα7-DN, the volume of VA6 also does not revert to that of unexposed flies (compare controls, Fig 4B, lanes 3 and 4, with flies expressing Dα7-DN, Fig 4C, lanes 11 and 12). This suggests that activation of the ACh receptor initiates a process that once initiated extends beyond the time of odor exposure.

### Notch in ORNs regulates the physiological plasticity of PNs solely by the canonical pathway

**Fig 5:**
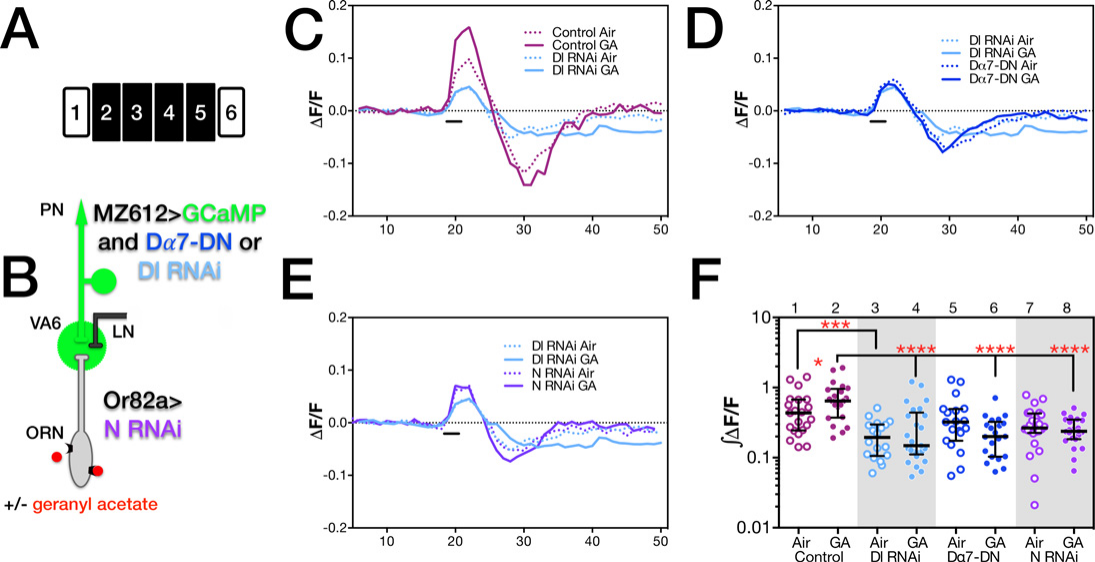
Delta in PNs is required for PN physiological plasticity. (**A**) Schematic of the experimental protocol. (**B**) The relevant genotypes are shown on the cartoon of the olfactory circuit. In **C** through **F** female flies were exposed to 1% GA in paraffin oil or paraffin oil alone and removed from odor for one day prior to imaging. The traces in **C**, **D** and **E** depict the median ΔF/F for each genotype and condition over time. (The numbers on the x-axis are frames of 472 msec each). The black bar indicates the time of a 1.5 second GA pulse. Solid traces, GA exposed flies; dotted trace, air exposed flies; magenta traces, control; pale blue traces, *Dl* RNAi; purple traces, *N* RNAi. (**F**) ∫ΔF/F, plotted in log scale, of calcium influx of flies shown in **C, D** and **E**. Statistically significant comparisons are indicated by asterisks. All other biologically relevant comparisons were not statistically significant. The number of quantified glomeruli are: lane 3, 18; lanes 4, 25; control and Dα7-DN flies are the same as in Fig 2E. Control, *Dl* RNAi and Dα7-DN experiments were carried out at the same time. In **D** the *Dl* RNAi (pale blue) and Dα7-DN (blue, data from Fig 2) are superimposed and in **E** traces of *Dl* RNAi in PNs (pale blue) are superimposed with those of *N* RNAi in ORNs (purple, data from Kidd et al, 2015 [10]). Flies were MZ612-GAL4 UAS.GCaMP6s/Or82a-LexAGAD; UAS.GCaMP6s LexOP.dsRED with UAS.Dl shRNA (*Dl* RNAi), with UAS.Dα7-DN (Dα7-DN) or with neither (control) and MZ612-GAL4 UAS.GCaMP6s/Or82a-LexAGAD; UAS.GCaMP6s LexOP.dsRED with LexOP.N shRNA (*N* RNAi, data from Kidd et al., 2015 [10]).

As described above and previously, when flies are chronically exposed to an odor, in addition to the odorant specific increase in the volume of glomeruli that respond to that odor, there is a change in the activity of neurons in the olfactory circuit in response to subsequent presentation of the same odor [6,8,10,23,26]. For geranyl acetate prior exposure leads to an increase in calcium influx into PN dendrites [10]. We have shown that Notch in ORNs, is required for both forms of plasticity. When regulating morphological plasticity Notch acts via both canonical and non-canonical mechanisms [10]. To address if this is also the case when regulating physiological plasticity, we used the GAL4 system to express *Delta* RNAi along with GCaMP6s [54] in VA6 PNs (Fig 5B). We exposed control flies and flies expressing *Delta* RNAi to air or geranyl acetate for 4 days, removed the flies from odor for one day, and using 2-photon microscopy measured calcium influx into VA6 PN dendrites upon subsequent presentation of geranyl acetate (Fig 5A).

Calcium influx into PN dendrites of flies expressing *Delta* RNAi differs from that of control flies in two ways. First, even in air exposed flies there is a decrease in calcium influx into PNs (compare dashed purple trace, control, with dashed blue trace, *Delta* RNAi, in the traces of the median ΔF/F in Fig 5C; and lanes 1 and 3 in the plots of ∫ΔF/F in Fig 5F). This indicates that even in the absence of prior odor exposure knocking down Delta in VA6 PNs affects their activation. We address this below. (We have shown previously that knocking down Delta in VA6 PNs does not affect the projection of the PNs to VA6 [10].) The second difference between control flies and flies expressing *Delta* RNAi is that unlike control flies, prior exposure of flies expressing *Delta* RNAi to geranyl acetate does not lead to an increase in calcium influx into PN dendrites (compare dashed and solid purple traces, controls, with dashed and solid blue traces, Delta RNAi, in Fig 5C; and the plots of ∫ΔF/F in Fig 5F, lanes 1 and 2, controls, versus lanes 3 and 4, Delta RNAi). Intriguingly, the calcium activity traces of PNs expressing *Delta* RNAi is similar to that of PNs expressing Dα7-DN (the traces of the median ΔF/F of *Delta* RNAi and Dα7-DN are superimposed in Fig 5D). We draw two conclusions about the differing roles of Notch and Delta from these observations. First, in regulating physiological plasticity Notch is only acting via the canonical pathway. Knocking down Delta in PNs has a similar phenotype as knocking down Notch in ORNs. This can be seen in Fig 5E, where the traces of the median ΔF/F of flies expressing *Delta* RNAi in PNs and *Notch* RNAi in ORNs (data from [10]) are superimposed. This is unlike what we observed when assaying morphological plasticity, where knocking down Delta in PNs and Notch in ORNs produced different phenotypes. Second, the relationship between morphological and physiological plasticity is not a straightforward one. Knocking down Notch in ORNs blocks both the enhanced activation of VA6 PNs upon subsequent exposure to geranyl acetate and the chronic odor induced increase in volume of VA6. However, whereas knocking down Delta in VA6 PNs blocks the enhanced activation of VA6 PNs upon subsequent exposure to geranyl acetate, the VA6 glomerulus still increases in volume when flies are chronically exposed to geranyl acetate. In fact the volume increase is larger than that of wild type flies [10]. We might have predicted therefore that knocking down Delta would lead to an even greater increase in calcium activity in PNs than is observed in control flies. This is not however what we observed.

### Expression of NICD in ORNs results in a prolonged increase in calcium levels in PNs

**Fig 6:**
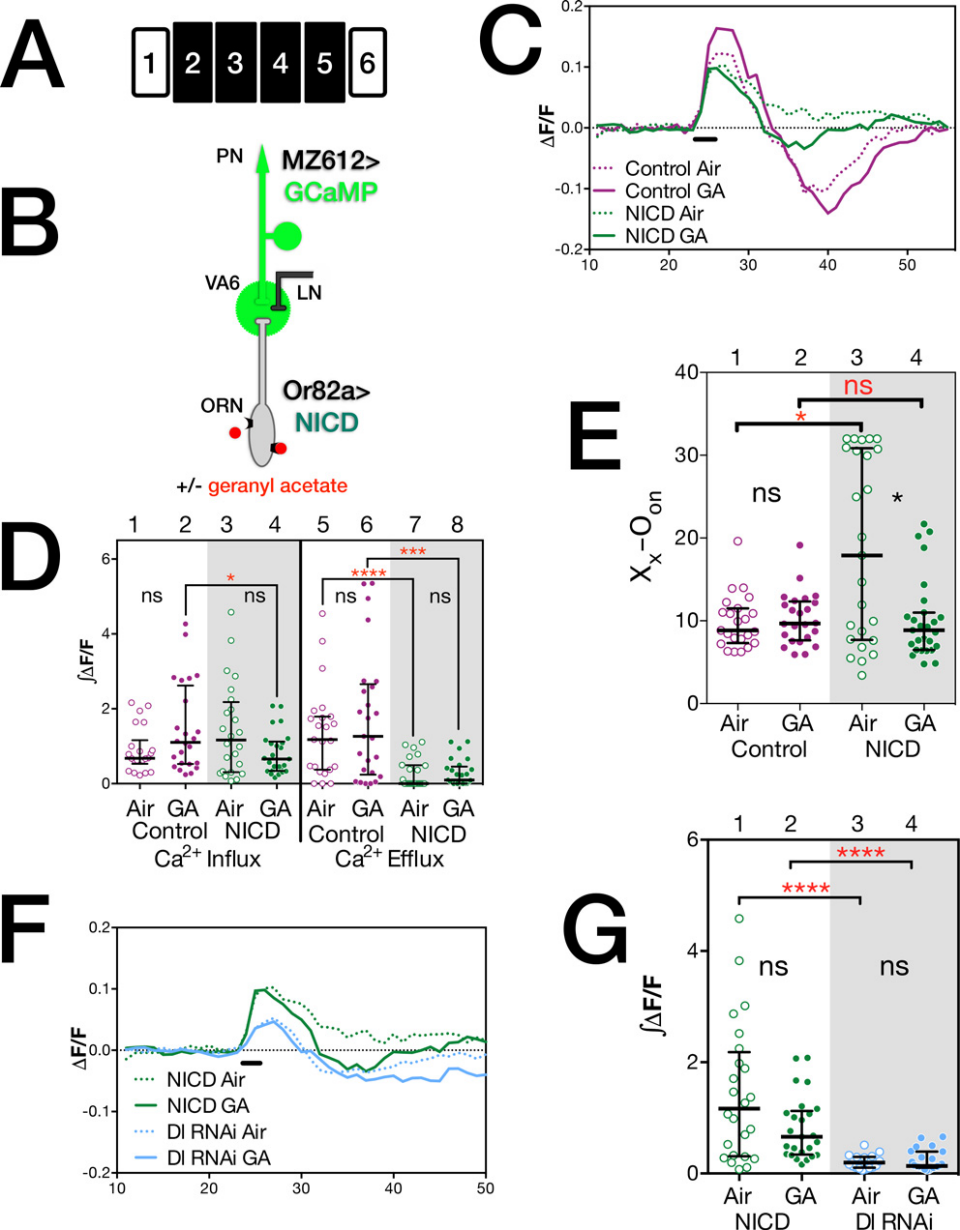
Expression of NICD in ORNs results in a prolonged increase in calcium levels in PNs. (**A**) Schematic of the experimental protocol. (**B**) The relevant genotypes are shown on the cartoon of the olfactory circuit. In **C** through **G** female flies were exposed to 1% GA in paraffin oil or paraffin oil alone and removed from odor for one day prior to imaging. The traces in **C** and **F** depict the median ΔF/F for each genotype and condition over time. (The numbers on the x-axis are frames of 472 msec each). The black bar indicates the time of a 1.5 second GA pulse; solid traces, GA exposed flies; dotted traces, air exposed flies; magenta traces and circles, control; green traces and circles, NICD; pale blue traces and circles, *Dl* RNAi. (**D**) ∫ΔF/F, of calcium influx and efflux of flies shown in **C**. The statistics are based on the following number of glomeruli: lanes 1 and 5, 22; lanes 2 and 6, 24; lanes 3 and 7, 24; lanes 4 and 8, 25. (**G**) ∫ΔF/F of calcium influx of flies plotted in **F**. (**E**) The number of frames between odor onset (O_on_) and the end of calcium influx X_x._ Open circles air exposed, filled circles GA exposed flies. The statistics are based on the following number of glomeruli: lane 1, 23; lane 2, 24; lane 3, 25; lane 4, 27. Flies were MZ612-GAL4 UAS.GCaMP6s/Or82a-LexAGAD; UAS.GCaMP6s LexOP.dsRED carrying either LexOP.mCherry shRNA (control) or LexOP.NICD (NICD) and MZ612-GAL4 UAS.GCaMP6s/Or82a-LexAGAD; UAS.GCaMP6s LexOP.dsRED with UAS.Dl shRNA (*Dl* RNAi, data from Fig 5).

Both knocking down Notch in VA6 ORNs and knocking down Delta in VA6 PNs blocked the enhanced activation of VA6 PNs that is observed when geranyl acetate is presented to control flies that had been chronically exposed to geranyl acetate. Thus, Notch appears to be regulating physiological plasticity solely by the canonical Notch pathway. In the canonical Notch pathway, Delta binding to Notch induces a series of proteolytic cleavages that result in the cytoplasmic domain of Notch (NICD), in association with its transcriptional effector Suppressor of Hairless (Su(H)), entering the nucleus and activating transcription (Fig 1A(i); reviewed in [43]). Over-expression of NICD could be expected to reveal a mechanism that might not be detectable using our assays due to the low level of NICD produced in wild type flies upon Notch activation and the lack of signal amplification ([60–62], reviewed in [43]). We therefore asked what effect expressing NICD in VA6 ORNs has on the activation of VA6 PNs when air exposed flies or flies that had been chronically exposed to geranyl acetate, are transiently presented with geranyl acetate (Fig 6A). To do this we used a GAl4 driver to express GCaMP6s in VA6 PNs and Or82a-LexA-GAD to express NICD in VA6 ORNs (Fig 6B). The traces of ΔF/F are presented in Fig 6C. In air exposed control flies, following odor offset, the level of calcium decreases to below that found prior to odor onset (Fig 6C, dotted purple trace). This reflects suppression of PN activity below spontaneous levels. (ORNs are noisy and fire in the absence of odor, and depending on the class of ORN, following odor offset, ORN and consequently PN activity can be suppressed below spontaneous levels (reviewed in [22]).) In air exposed flies expressing NICD in VA6 ORNs, this enhanced efflux is not observed (Fig 6C, dotted green trace). The difference in efflux is also evident in the plots of ∫ΔF/F in Fig 6D (compare lanes 5 and 7). That the calcium responses of air exposed control and NICD expressing flies are different can also be seen by comparing the number of frames between odor onset (O_on_) and the end of calcium influx (X_x_; Fig 6E, lanes 1 and 3). These data indicate that expression of NICD in VA6 ORNs leads to a prolonged influx of calcium into VA6 PN dendrites, perhaps due to prolonged ACh release by ORNs, as we discuss in the next section.

In prior experiments chronically exposing control flies to geranyl acetate led to an increase in calcium influx into PN dendrites when the flies were later exposed to a transient pulse of geranyl acetate (Fig 2D and 2E, and [10]). While the calcium responses of control and odor exposed flies are not significantly different in the experiment presented here, the trend is in the same direction, as can be seen by comparing the dotted and solid purple traces in Fig 6C, and lanes 1 and 2 in the plots of ∫ΔF/F in Fig 6D. This trend is not observed in flies expressing NICD. (Compare dotted green, air exposed, and solid green, geranyl acetate exposed traces in Fig 6C; and lanes 3 and 4 in the plots of ∫ΔF/F in Fig 6D). Instead, prior exposure of flies expressing NICD to geranyl acetate resulted in a decrease in the number of frames between odor onset and the end of calcium influx (Fig 6E, lanes 3 and 4). We attribute this to the combination of long-term odor exposure and the expression of NICD resulting in depletion of ACh. We will address this further below.

In Fig 6F and 6G we compare the effect of knocking down Delta in PNs with expressing NICD in ORNs on calcium activation in PNs. As is to be expected if Notch affects PN activation solely via the canonical pathway, and Delta in PNs activates Notch in ORNs, the expression of NICD in ORNs leads to enhanced calcium activity compared to the expression of *Delta* RNAi in PNs. This can be seen both in the traces of ΔF/F (Fig 6F; NICD, green traces; *Delta* RNAi, pale blue traces) and the plot of ∫ΔF/F (Fig 6G; NICD, green circles; *Delta* RNAi, pale blue circles).

### Exposing flies expressing NICD in ORNs to geranyl acetate leads to an increase in ChAT in VA6

**Fig 7:**
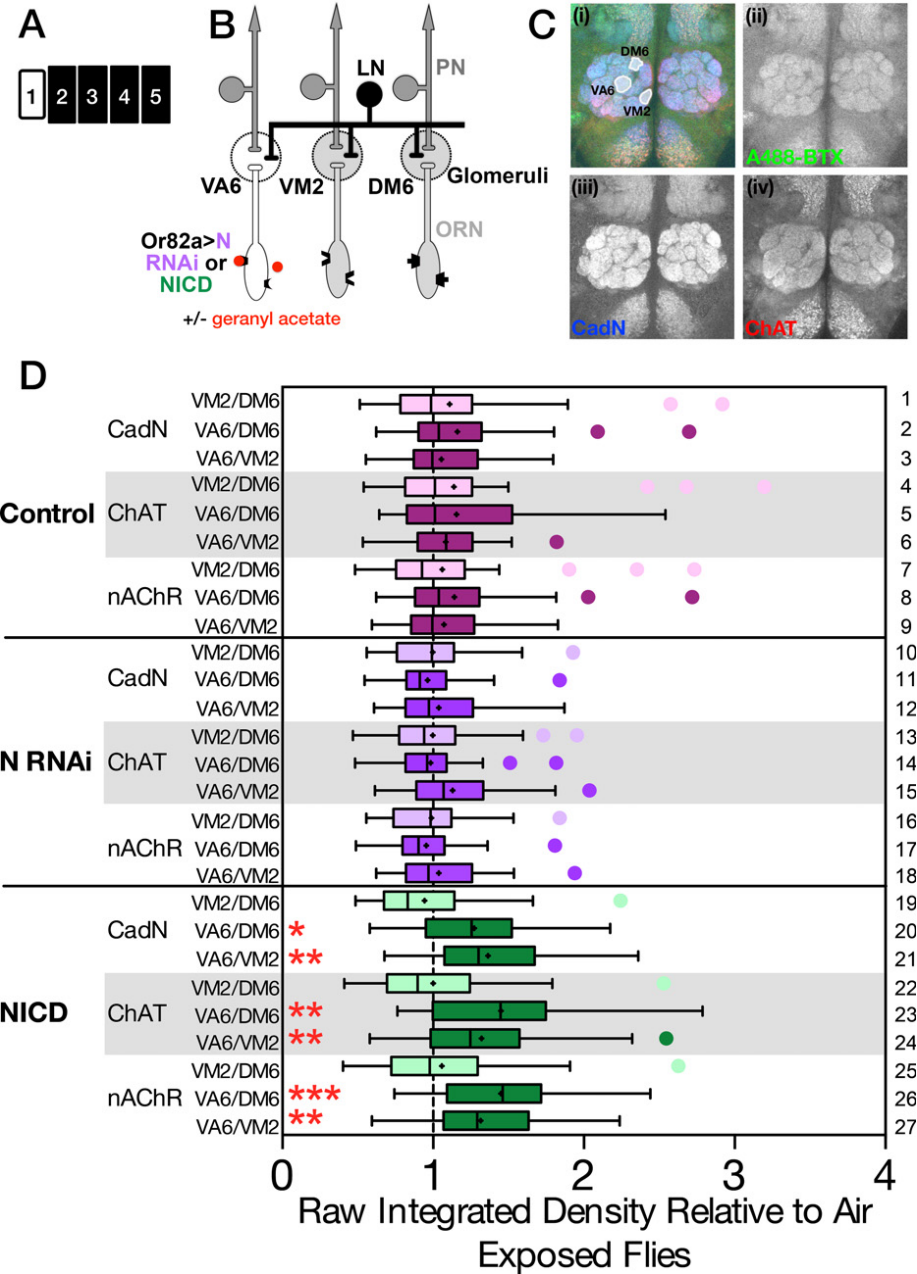
Chronically exposing flies expressing NICD in ORNs to geranyl acetate leads to an increase in ChAT in VA6. (**A**) Schematic of the experimental protocol. (**B**) The relevant genotypes and glomeruli are shown on the cartoon of the olfactory circuit. (**C**) Female flies were exposed to 1% GA in paraffin oil or paraffin oil alone for four days. Brains were then co-reacted with anti-cadN, anti-ChAT and A488-BTX to visualize nAChRs. A representative brain is shown in **C(i)** with the three glomeruli which were quantified outlined. Grey scale images of each channel are also shown separately: (**ii**) A488-BTX (green), (**iii**) cadN (blue), and (**iv**) ChAT (red). (**D**) For each brain we quantified the pixel intensities of VA6, VM2 and DM6 and then determined the ratios of all three combinations. Each ratio for geranyl acetate and air exposed flies was normalized to the median value for air exposed flies. Data for the geranyl acetate exposed flies are shown as box and whisker plots. The box represents the median and inner quartiles, the whiskers the outer quartiles. The cross is the mean, and outliers are shown as circles. Normalized air exposed flies by definition have a median of one, which is indicated by the dashed line. The complete data sets of unnormalized and normalized ratios are shown in the scatter plots of S1 Fig. Statistical significance was determined by the Kruskal-Wallis test with Dunn’s correction for multiple comparisons. Control flies are magenta, *N* RNAi are purple and NICD flies green. The control comparisons of VM2/DM6 are more lightly shaded than the experimental VA6/DM6 and VA6/VM2 comparisons. Flies were Or82a-GAL4 with either UAS.Val20, UAS.N shRNA or UAS.NICD.

Given that ORN to PN synapses are cholinergic, the observation that manipulating components of the Notch pathway affected PN activation led us to hypothesize that Notch activation affects the level of ACh released by ORNs. As a first test of this possibility, we asked whether the expression of *Notch* RNAi or NICD in VA6 ORNs affected the level of Choline acetyltransferase (ChAT, which catalyzes the synthesis of ACh) in VA6. Control flies and flies expressing *Notch* RNAi or NICD in VA6 ORNs (Fig 7B) were exposed to air or geranyl acetate for 4 days (Fig 7A), and for each genotype and condition the level of ChAT in VA6 (as detected with anti-ChAT antisera) was compared with the level of ChAT in 2 glomeruli that are not activated by geranyl acetate (VM2 and DM6). As a further control for this experiment, we also compared the levels of ChAT between the 2 glomeruli that do not respond to geranyl acetate. A representative antennal lobe reacted with anti-ChAT antisera is shown in Fig 7C(iv) and the glomeruli quantified are highlighted in Fig 7C(i). (The brains were simultaneously also reacted with anti-cadN and alpha-Bungarotoxin-A488 to visualize nAChRs. The merged image is in Fig 7C(i)). For each genotype the ChAT ratios (VA6/DM6, VA6/VM2 and VM2/DM6) were normalized to the air exposed flies. The ratios of the odor exposed flies are plotted in Fig 7D. The median of the ratios of the air exposed flies is by definition 1 and is denoted by the dashed line in the figure. (The entire data set is presented in S1 Fig) In flies expressing NICD in VA6 ORNs, chronic exposure to geranyl acetate resulted in an increase in the level of ChAT in VA6 (Fig 7D, lanes 22–24, 1.4× for the VA6/DM6 comparison and 1.2× for the VA6/VM2 comparison). This increase is dependent upon odor exposure, as in air exposed flies the expression of NICD does not lead to an increase in ChAT in VA6 (S1 Fig, compare air exposed controls (S1A Fig, lane 3) with air exposed NICD flies (S1A Fig, lane 15). Importantly, the observation that the increase requires odor suggests that it is not merely an artifact caused by over-expression of NICD. Exposing control flies to geranyl acetate did not lead to an increase in ChAT levels (Fig 7D, lanes 4–6). We will address this in the Discussion below. We did not observe a decrease in ChAT in *Notch* RNAi flies (Fig 7D, lanes 13–15) either because the change is below the level of detection of our assay, or because Notch does not regulate that basal level of ChAT.

In a similar fashion, we also assayed the level of nicotinic ACh receptors (nAChRs; as detected with Alexa Fluor 488 α-bungarotoxin, A488-BTX) in the same brains. A gray scale image of the antennal lobe reacted with A488-BTX is shown in Fig 7C(ii). In addition to leading to an increase in ChAT in VA6, exposing flies expressing NICD to geranyl acetate also resulted in an increase in the level of nAChRs in VA6 (Fig 7D, lanes 25–27, 1.4× for the VA6/DM6 comparison and 1.3× for the VA6/VM2 comparison). As we observed for ChAT, the increase in level of of nAChRs seen in flies expressing NICD is dependent upon odor (S1 Fig, A lanes 5 and 17).

The antennal lobes were also reacted with an antibody (DN-Ex) directed against Cadherin-N (CadN) a homophilic cell adhesion molecule [63]. This antisera labels all neuropiles (Fig 7C(iii)) [64]. In flies expressing NICD, exposure to geranyl acetate also led to an increase in the level of CadN in VA6 (Fig 7D, lanes 19–21, 1.2× for the VA6/DM6 comparison and 1.3× for the VA6/VM2 comparison). Increased neuronal activity has been shown to increase CadN levels [65].

## Discussion

**Fig 8:**
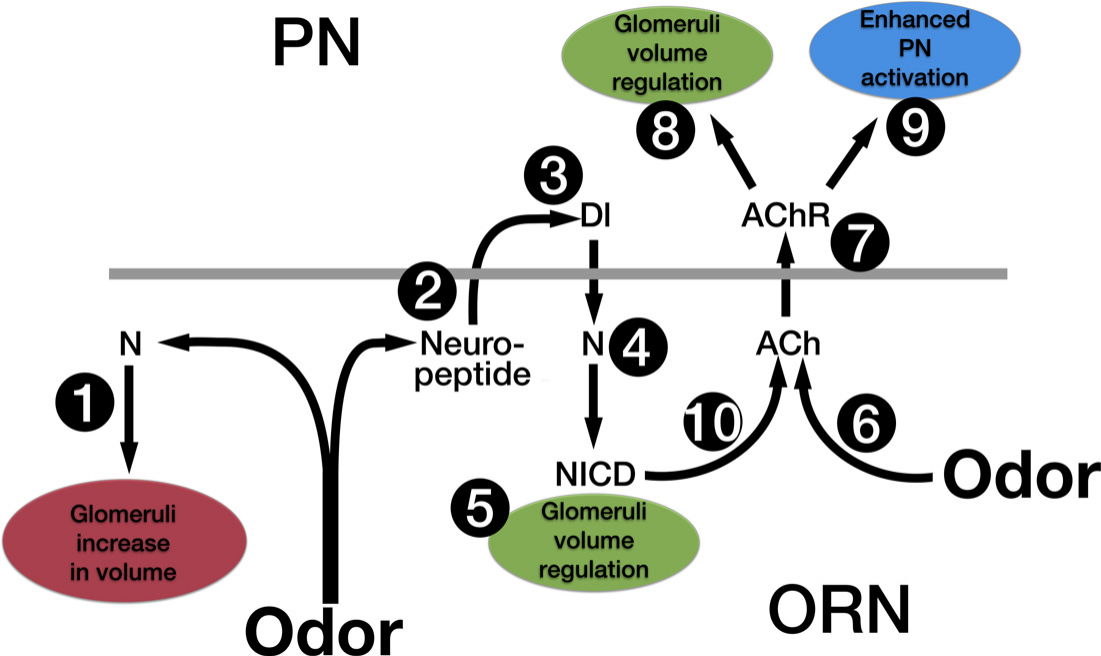
A model for how Notch in ORNs, in conjunction with chronic odor exposure, regulates olfactory plasticity. Based on the results described in this paper we propose that upon chronic odor exposure, N, via a non-canonical mechanism, induces an increase in glomeruli volume (1). Chronic odor also causes the release of a neuropeptide from ORNs (2) that activates Dl in PNs (3). Dl in PNs activates the canonical N pathway in ORNs (4) to regulate glomeruli volume (5). Activation of the canonical N pathway in conjunction with long-term odor exposure (6) leads to an increase in activation of nAChRs (7). This has two effects: it activates a mechanism in PNs that can also regulate the increase in glomeruli volume (8), and it enhances PN activation (9). We hypothesize that both the canonical N pathway (10) and chronic odor (6) lead to an increase in ACh release from ORNs.

We previously established that odor dependent Notch activation in ORNs mediates structural and physiological responses to prolonged odor exposure [10]. Here we set out to further characterize how this occurs. Our data are summarized in the model depicted in Fig 8. Below we discuss our results in the context of this model.

### Notch and the regulation of morphological plasticity

In regulating the odor induced increase in glomerular volume we previously demonstrated that Notch functions by both non-canonical and canonical mechanisms. Non-canonical Notch signaling is required for the increase in volume, and canonical Notch signaling regulates the extent of the increase (Fig 1C) [10]. Here we have shown that glomeruli still increase in volume despite vesicle release by ORNs being blocked by the expression of tetanus toxin (Fig 3C, lanes 7 and 8). This suggests that one mechanism by which glomeruli increase in volume is autonomous to ORNs. Because a non-canonical Notch pathway is required in ORNs for the odor induced increase in volume, this also suggests that the mechanism by which the non-canonical Notch pathway mediates the increase in volume is autonomous to the ORN (Fig 8, #1).

In fact, in flies expressing tetanus toxin in ORNs, the increase in volume is larger than that of control flies (Fig 3C, lanes 6 and 8). Hence, unlike the increase in volume, regulation of the extent of the volume increase does require communication with downstream neurons. We previously showed that the regulation of glomerular volume also requires Delta in PNs [10] and that expression of tetanus toxin in ORNs blocks the activation of the canonical Notch pathway by Delta [51]. These observations led us to hypothesize that Delta activation required cholinergic activation of PNs [10]. However, knocking down the function of nAChRs by the expression of Dα7-DN in PNs did not block activation of the canonical Notch pathway (Fig 2F). We therefore suggest that Delta upon long-term odor exposure is activated by the release from ORNs of a neuropeptide rather than a neurotransmitter (Fig 8, #2). Tetanus toxin has also been shown to affect neuropeptide release [55,56]. The *Drosophila* genome contains at least 42 genes that encode neuropeptides, peptide hormones or protein hormones of which at least 16 appear to be expressed in adult antennae [66–68].

Expression of Dα7-DN in PNs also results in a greater increase in glomerular volume than is observed in control flies (Fig 3C, lanes 2 and 4). Thus, knocking down the function of nAChRs has the same phenotype as knocking down Delta, yet knocking down the function of nAChRs does not affect activation of the Notch reporter (Fig 2F), indicating that activation of nAChRs does not activate Delta. This argues that the canonical Notch pathway, and ACh mediated PN activation, regulate the extent of the odor induced increase in glomerular volume by parallel pathways (Fig 8, #5 and #8). We therefore suggest that in addition to the regulation of glomerular volume that occurs in ORNs and is mediated by the canonical Notch pathway, there is a mechanism in PNs that also regulates the extent of the increase in glomerular volume, and this mechanism in PNs requires cholinergic activation of PNs (Fig 8, #7). As we discuss in more detail below, Delta activation of the canonical Notch pathway is upstream of nAChR activation (Fig 8, #4 and #7). It is therefore possible that the mechanism that regulates the extent of the odor induced increase in glomerular volume only exists in PNs. However, it is not unexpected that changes in the morphology of glomeruli would require there to be coordination of structural changes in all type of neurons that innervate the glomerulus. For example, Sachse et al. hypothesized that long-term odor exposure led to neuroanatomical changes in LNs [6].

Das et al. have shown that long-term odor induced structural plasticity requires *rutabaga* encoded adenylate cyclase and the vesicular glutamate transporter (DVGLUT) in LNs and N-Methyl-D-aspartate (NMDA) receptors in PNs [69]. Our work showing that Notch is required in ORNs for changes in glomerular volume induced by long-term exposure to either aversive (CO_2_) or attractive (geranyl acetate) odors does not conflict with these observations, but rather adds an additional aspect to the regulation of structural plasticity. However the findings of Das et al. are difficult to reconcile with our experiments showing that glomeruli still increase in volume despite vesicle release by ORNs being blocked by the expression of tetanus toxin (Fig 3C). One possible explanation could be that odor alone or odor activating the non-canonical Notch pathway induces the release from ORNs of a factor not blocked by the expression of tetanus toxin. This could be tested by epistasis experiments; for instance assaying the effect on structural plasticity of expressing tetanus toxin in ORNs or knocking down Delta in PNs in flies that are also mutant for *rutabaga*. As mentioned above Sachse et al. found that the increase in volume of V upon long-term exposure to CO_2_ was not due to changes in the morphology of V ORNs and hypothesized that long-term odor exposure led to neuroanatomical changes in LNs [6]. However, whereas Sachse et al. measured the volume of the entire glomerulus, including area not labeled by GFP, we measured only the volume occupied by the ORNs.

### Notch and the regulation of physiological plasticity

In control flies prior long-term exposure to the attractive odor geranyl acetate resulted in enhanced PN activation upon subsequent presentation of geranyl acetate (Fig 2D and 2E and [10]). This is in contrast to the decreased PN activation previously observed in response to long-term exposure to the repulsive odors CO_2_ or ethyl butyrate [6,69]. When assaying physiological plasticity, we exposed flies to geranyl acetate on food. Flies were then removed from odor and starved for one day prior to imaging. Unlike the decrease in behavioral responsiveness previously observed to CO_2_ and ethyl butyrate [6,69], our exposure paradigm to geranyl acetate resulted in an increase in behavioral attraction [10]. This suggests that unlike non-associative conditioning paradigms previously used [6,69], we are using an associative conditioning paradigm analogous to the “imaginal conditioning” described by Siddiqi and co-workers [9].

The geranyl acetate induced enhanced PN activation does not occur when Notch is knocked down in ORNs [10] or when Delta is knocked down in PNs (Fig 5C and 5D). This indicates that the enhanced projection neuron activation requires the canonical Notch pathway. In addition, knocking down the activity of nAChRs in PNs also blocks the odor induced enhanced PN activation (Fig 2D and 2E).

Chronically exposing flies expressing NICD in VA6 ORNs to geranyl acetate leads to an increase in the amount of ChAT in VA6 (Fig 7D, lanes 22–24). Because in the canonical Notch pathway, Notch is a transcription factor, it is possible that ChAT is being directly upregulated by Notch in ORNs. Alternatively, the effect of NICD on ChAT levels could be indirect resulting from a depletion in ACh induced by exposing flies expressing NICD to geranyl acetate. Because chronically exposing control flies to odor leads to an increase in PN activation (Fig 2D and 2E and [10]) and the over-expression of NICD leads to prolonged PN activation (Fig 6C and 6E), we speculate that both exposure to odor and the expression of NICD on their own lead to enhanced ACh release from ORNs. However, only in combination is the amount of Ach released sufficient to necessitate an increase in ChAT. Our observation that neither the expression of NICD in the absence of prior odor exposure (S1 Fig, A lanes 3 and 15), nor the exposure of control flies to odor (Fig 7D, lanes 4–6) leads to an increase in ChAT, indicates that the effects are synergistic (Fig 8, #6 and #10). In the canonical Notch pathway, Delta binding to Notch results in the cleavage of Notch and the release of the cytoplasmic domain (NICD). We propose that activation of the canonical Notch pathway that occurs as a consequence of long-term odor exposure (Fig 8, #4 and #10) in conjunction with a parallel pathway that is also induced by long-term odor exposure (Fig 8, #6) results in enhanced Ach release from ORNs and enhanced PN activation. (Fig 8, #9). If the synergistic effect of prior odor exposure and the expression of NICD leads to a depletion of ACh, a homeostatic mechanism could lead to an increase in the level of nAChRs (Fig 7D, lanes 25–27) (reviewed in [70]).

This model also explains why prior exposure of flies expressing NICD to odor suppresses the prolonged influx of calcium into VA6 PN dendrites (Fig 6C and 6E). Prior odor exposure in the context of the expression of NICD leads to a depletion of ACh and therefore the prolonged influx no longer occurs. Exposing control flies to geranyl acetate did not lead to an observable increase in ChAT levels (Fig 7D, lanes 4–6), either because the increase is below the level of detection of our assay or because there is still sufficient ACh.

Knocking down Delta in PNs affects the calcium activity in PNs not only of flies that had been chronically exposed to geranyl acetate, but also of air exposed flies (Fig 5C and 5F). We expose flies to odor on food, and there is an odor in the food that activates VA6 ORNs [51]. Thus, the pathway that activates Delta is functioning, albeit to a lesser extent, in air exposed flies, and it is therefore not unexpected that knocking down Delta would have an effect on PN activation in air exposed flies.

### The role of Lqf/Epsin in non-canonical Notch signaling

Lqf mediates Delta internalization, which is required for Delta activation of the canonical Notch pathway [57,58]. Our data show that in the absence of Lqf, non-canonical Notch signaling, which is Delta independent and does not require PN activation, is also inhibited (Fig 3D). This inhibition is relieved by simultaneously knocking down Delta; the glomeruli again increase in size (Fig 3D), indicating that the activity of the non-canonical Notch pathway has been restored. We interpret these results as indicating that in the presence of odor and in the absence of Lqf, Delta in PNs forms a non-productive complex across the synapse with Notch in ORNs. The non-productive Delta/Notch interaction sequesters Notch away from components of the non-canonical Notch pathway blocking the increase in glomerular volume. These components could include another ligand on PNs or proteins in the ORNs. This instance of Delta inhibiting Notch is unusual. Previously described mutual inhibition of Notch and Delta occurs when Notch and Delta are expressed on the same cell; the inhibition is occurring in cis (reviewed in [71]). Here Delta and Notch are on separate cells, thus Delta is inhibiting Notch in trans. Such trans inhibition might only be observed in processes where non-canonical Notch signaling is involved.

We suggest that upon long-term odor exposure both non-canonical and canonical Notch signaling are activated to regulate glomerular volume, with the rate of the non-canonical pathway initially exceeding that of the canonical pathway, thus allowing for the increase in glomerular volume. The fact that knocking down Lqf completely blocks the increase in volume suggests that the inhibitory Notch/Delta complex is either preexisting or forms very rapidly upon odor exposure. Perhaps a Notch/Delta/Lqf complex exists at the synapse prior to odor exposure and Lqf ensures the complex is in an appropriate location to allow for odor induced non-canonical Notch signaling. Alternatively, the association of Lqf with Delta could be regulating both the spatial and temporal trafficking of Delta to the synapse. In the absence of Lqf, Delta traffics to the synapse prematurely, associates with Notch and blocks non-canonical signaling.

### Relationship between morphological and physiological plasticity

In flies, honeybees and mice it has been shown that odor exposure in the context of associative or non-associative conditioning leads to an increase in glomerular volume. We have shown that knocking down Notch in adult *Drosophila* ORNs prevents both the long-term odor induced increase in calcium activity in PNs and the increase in glomerular volume [10]. Knocking down Delta in PNs also prevents the long-term odor induced increase in calcium activity in PNs (Fig 5C and 5F), but the increase in glomerular volume is even larger than that observed in control flies [10]. Thus, there is a disconnect between the changes in morphology and physiology. These data question the directness of the connection between morphological and physiological plasticity.

These observations are in accord with work recently published by Morrison et al. [17]. They observed that olfactory fear conditioning leads to an odor specific increase in glomerular area, and that olfactory extinction specific to the conditioned odor reverses both the behavior and the change in the morphology of the glomerulus. However, in fear conditioned flies the acquisition of freezing behavior occurs prior to the increase in area, and a decrease in freezing can be observed despite there being no change in glomerular structure. There is a dissociation between behavioral and morphological plasticity. Similarly Kass et al. observed an increase in ORN output in the context of fear conditioning that was not associated with a change in glomerular area [15].

We previously demonstrated that Notch is required in ORNs for functional and morphological plasticity in response to chronic odor exposure. Our data point to a key role for Notch in mediating morphological plasticity at the first olfactory synapse in response to both repulsive and attractive odors. We have further demonstrated a role for Notch in mediating physiological plasticity in an associative conditioning paradigm that utilizes an attractive odor, and have suggested a mechanism by which this occurs. It would be of interest to determine if Notch plays a similar role in mediating plasticity in non-associative conditioning paradigms utilizing repulsive odors. Our work highlights that, as it does during development, Notch plays a central role in learning and memory.

## Materials and Methods

### Constructs and fly stocks

The following transgenic flies were generated: UAS.Val20: pVALIUM20 [72] was injected into attP2. LexOP.NICD: The UAS sequences in UAS.NICD [10] were replaced with the LexA operator sequences of pJFRC19-13XLexAop2-IVS-myr::GFP [73] Addgene plasmid 26224. UAS. Dα7-DN: the Dα7-DN sequence was isolated by PCR from flies containing UAS-Da7-Y195T [53] and subcloned as a BglII/NotI fragment into pValium20.

Other transgenic stocks were Or82a-GAL4 [74], UAS.mCD8-GFP [75], Or82a-CD8-GFP [59], UAS.N shRNA TRiP.HMS00015 [72], UAS.Dl shRNA TRiP.HMS01309, UAS.mCherry in attP2, 20XUAS.GCaMP6s in attP40 and VK00005 [54], N-LV [51], LexOP.dGFP [51], Or82a-LexA-GAD [10], LexOP.dsRED [10], MZ612-GAL4 [76], UAS.IMPTNT and UAS.TNT [77], tubP-gal80^ts^ [78], UAS.espin RNAi [10], UAS-Dcr-2.D [79].

### Odorant Exposure

One milliliter of odorant in paraffin oil was placed in a 1.7 ml microcentrifuge tube covered with Nitex. The microcentrifuge tube, along with approximately 30–40 flies, was placed into a 25 × 95 mm polystyrene *Drosophila* vial containing approximately 10 ml of dextrose food [51].

### Measurement of glomerular volume

Volume measurements were determined as described previously [10], except that dsRed was visualized directly, without the use of an antibody. All experiments were carried out blind.

### 2-photon image analysis

Flies were prepared for 2-photon microscopy, odor was delivered, and imaging performed as described [10]. Rather than manually removing aberrant traces, for inclusion in the ∫ΔF/F scatterplots and subsequent analysis, peaks had to be at least 10% of the maximum Y value and ≥ 3 adjacent point on X axis. Outliers were removed by the ROUT method [80] described at http://www.graphpad.com/guides/prism/6/statistics/index.htm?stat_how_to_removing_outliers.htm. Figs 2, 5 and 6G Q = 0.1% (removes definitive outliers); Fig 6D and 6E, Q = 2% (98% of removed outliers are actual outliers).

### Notch reporter assays

N reporter activity was determined as described previously [51].

### ChAT, nAChR and CadN immunofluorescence

Brains were dissected and fixed as described by Pfeiffer et al. [73]. Brains were reacted with ChAT4B1 and DN-Ex #8 (both from Developmental Studies Hybridoma Bank) to detect ChAT and CadN), followed by incubation with Cy3 conjugated anti-mouse and Cy5 conjugated anti-rat F(ab')_2_ fragments (both from Jackson ImmunoResearch). After the final wash, they were rinsed 3 × 5 min in PBS with 0.1% Triton X-100 at room temp, incubated for 2 hr with 1:500 α-Bungarotoxin, Alexa Fluor 488 conjugate (ThermoFisher Scientific) in PBS 0.1% TX-100, rinsed 3 × 5min with PBS 0.1% TX-100, mounted in SlowFade Diamond Antifade Mountant (ThermoFisher Scientific) and visualized immediately. All experiments were carried out blind.

## Supporting Information

S1 Fig Exposing flies expressing NICD in ORNs to geranyl acetate leads to an increase in ChAT in VA6. Female flies expressing *N* RNAi or NICD in VA6 ORNs, or control flies, were exposed to 1% GA in paraffin oil or paraffin oil alone for four days. Brains were then co-reacted with anti-cadN, anti-ChAT and A488-BTX to visualize nAChRs. (**A**) For each brain we quantified the pixel intensities of VA6, VM2 and DM6 and then determined the ratios of all three combinations. The ratios are presented as scatter plots. (**B**) Each ratio for GA and air exposed flies was normalized to the median value of the air exposed flies. The ratios are presented as scatter plots. Normalized air exposed flies by definition have a median of one, which is indicated by the dashed line. Statistical significance was determined by the Kruskal-Wallis test with Dunn’s correction for multiple comparisons. Controls are in magenta, *N* RNAi are purple and NICD are green. Open circles are air exposed and filled circles are geranyl acetate exposed flies. Flies were Or82a-GAL4 with either UAS.Val20, UAS.N shRNA or UAS.NICD.
(TIF)

## References

[1] Li Q, Liberles SD. Aversion and Attraction through Olfaction Review. Curr Biol. Elsevier Ltd; 2015;25: R120–R129. 10.1016/j.cub.2014.11.044 PMC4317791 25649823

[2] Twick I, Lee JA, Ramaswami M. Olfactory habituation in Drosophila-odor encoding and its plasticity in the antennal lobe. Prog Brain Res. 2014;208: 3–38. 10.1016/B978-0-444-63350-7.00001-2 24767477

[3] Busto GU, Cervantes-Sandoval I, Davis RL. Olfactory learning in Drosophila. Physiology (Bethesda). 2010;25: 338–346. 10.1152/physiol.00026.2010 21186278 PMC3380424

[4] Devaud JM, Acebes A, Ferrús A. Odor exposure causes central adaptation and morphological changes in selected olfactory glomeruli in Drosophila. J Neurosci. 2001;21: 6274–6282. 11487650 10.1523/JNEUROSCI.21-16-06274.2001 PMC6763130

[5] Devaud JM, Acebes A, Ramaswami M, Ferrús A. Structural and functional changes in the olfactory pathway of adult Drosophila take place at a critical age. J Neurobiol. 2003;56: 13–23. 10.1002/neu.10215 12767029

[6] Sachse S, Rueckert E, Keller A, Okada R, Tanaka NK, Ito K, et al Activity-dependent plasticity in an olfactory circuit. Neuron. 2007;56: 838–850. 10.1016/j.neuron.2007.10.035 18054860

[7] Arenas A, Fernández VM, Farina WM. Associative learning during early adulthood enhances later memory retention in honeybees. PLoS ONE. 2009;4: e8046 10.1371/journal.pone.0008046 19956575 PMC2779852

[8] Arenas A, Giurfa M, Farina WM, Sandoz JC. Early olfactory experience modifies neural activity in the antennal lobe of a social insect at the adult stage. Eur J Neurosci. 2009;30: 1498–1508. 10.1111/j.1460-9568.2009.06940.x 19821839

[9] Chakraborty TS, Goswami SP, Siddiqi O. Sensory correlates of imaginal conditioning in Drosophila melanogaster. J Neurogenet. 2009;23: 210–219. 10.1080/01677060802491559 19058083

[10] Kidd S, Struhl G, Lieber T. Notch Is Required in Adult Drosophila Sensory Neurons for Morphological and Functional Plasticity of the Olfactory Circuit. Desplan C, editor. PLoS Genet. 2015;11: e1005244 10.1371/journal.pgen.1005244.s006 26011623 PMC4444342

[11] Choi GB, Stettler DD, Kallman BR, Bhaskar ST, Fleischmann A, Axel R. Driving opposing behaviors with ensembles of piriform neurons. Cell. 2011;146: 1004–1015. 10.1016/j.cell.2011.07.041 21925321 PMC3230930

[12] Gore F, Schwartz EC, Brangers BC, Aladi S, Stujenske JM, Likhtik E, et al Neural Representations of Unconditioned Stimuli in Basolateral Amygdala Mediate Innate and Learned Responses. Cell. 2015;162: 134–145. 10.1016/j.cell.2015.06.027 26140594 PMC4526462

[13] Spors H, Albeanu DF, Murthy VN, Rinberg D, Uchida N, Wachowiak M, et al Illuminating vertebrate olfactory processing. J Neurosci. 2012;32: 14102–14108. 10.1523/JNEUROSCI.3328-12.2012 23055479 PMC3752119

[14] Guven-Ozkan T, Davis RL. Functional neuroanatomy of Drosophilaolfactory memory formation. Learning & Memory. 2014;21: 519–526. 10.1101/lm.034363.114 25225297 PMC4175493

[15] Kass MD, Rosenthal MC, Pottackal J, McGann JP. Fear Learning Enhances Neural Responses to Threat-Predictive Sensory Stimuli. Science. 2013;342: 1389–1392. 10.1126/science.1244916 24337299 PMC4011636

[16] Abraham NM, Vincis R, Lagier S, Rodriguez I, Carleton A. Long term functional plasticity of sensory inputs mediated by olfactory learning. eLIFE. 2014;3: e02109–e02109. 10.7554/eLife.02109.011 24642413 PMC3953949

[17] Morrison FG, Dias BG, Ressler KJ. Extinction reverses olfactory fear-conditioned increases in neuron number and glomerular size. Proceedings of the National Academy of Sciences. 2015;: 201505068 10.1073/pnas.1505068112 PMC4611645 26420875

[18] Ko KI, Root CM, Lindsay SA, Zaninovich OA, Shepherd AK, Wasserman SA, et al Starvation promotes concerted modulation of appetitive olfactory behavior via parallel neuromodulatory circuits. eLIFE. 2015;4 10.7554/eLife.08298 PMC4531282 26208339

[19] Vosshall LB, Amrein H, Morozov PS, Rzhetsky A, Axel R. A spatial map of olfactory receptor expression in the Drosophila antenna. Cell. 1999;96: 725–736. 10089887 10.1016/s0092-8674(00)80582-6

[20] Gao Q, Yuan B, Chess A. Convergent projections of Drosophila olfactory neurons to specific glomeruli in the antennal lobe. Nat Neurosci. 2000;3: 780–785. 10.1038/77680 10903570

[21] Hallem EA, Carlson JR. Coding of odors by a receptor repertoire. Cell. 2006;125: 143–160. 10.1016/j.cell.2006.01.050 16615896

[22] Wilson RI. Early Olfactory Processing in Drosophila: Mechanisms and Principles. Annu Rev Neurosci. 2013;36: 217–241. 10.1146/annurev-neuro-062111-150533 23841839 PMC3933953

[23] Das S, Sadanandappa MK, Dervan A, Larkin A, Lee JA, Sudhakaran IP, et al PNAS Plus: Plasticity of local GABAergic interneurons drives olfactory habituation. Proceedings of the National Academy of Sciences. 2011;108: E646–E654. 10.1073/pnas.1106411108 PMC3169145 21795607

[24] Jones SV, Choi DC, Davis M, Ressler KJ. Learning-dependent structural plasticity in the adult olfactory pathway. J Neurosci. 2008;28: 13106–13111. 10.1523/JNEUROSCI.4465-08.2008 19052201 PMC2613972

[25] Hourcade B, Perisse E, Devaud JM, Sandoz J-C. Long-term memory shapes the primary olfactory center of an insect brain. Learning & Memory. 2009;16: 607–615. 10.1101/lm.1445609 19794186

[26] Arenas A, Giurfa M, Sandoz JC, Hourcade B, Devaud JM, Farina WM. Early olfactory experience induces structural changes in the primary olfactory center of an insect brain. Eur J Neurosci. 2012;35: 682–690. 10.1111/j.1460-9568.2012.07999.x 22300014

[27] Costa RM, Honjo T, Silva AJ. Learning and memory deficits in Notch mutant mice. Curr Biol. 2003;13: 1348–1354. 12906797 10.1016/s0960-9822(03)00492-5

[28] Ge X, Hannan F, Xie Z, Feng C, Tully T, Zhou H, et al Notch signaling in Drosophila long-term memory formation. Proc Natl Acad Sci USA. 2004;101: 10172–10176. 10.1073/pnas.0403497101 15220476 PMC454384

[29] Presente A, Boyles RS, Serway CN, de Belle JS, Andres AJ. Notch is required for long-term memory in Drosophila. Proc Natl Acad Sci USA. 2004;101: 1764–1768. 10.1073/pnas.0308259100 14752200 PMC341850

[30] Wang Y, Chan SL, Miele L, Yao PJ, Mackes J, Ingram DK, et al Involvement of Notch signaling in hippocampal synaptic plasticity. Proc Natl Acad Sci USA. 2004;101: 9458–9462. 10.1073/pnas.0308126101 15190179 PMC438998

[31] Conboy L, Seymour CM, Monopoli MP, O'Sullivan NC, Murphy KJ, Regan CM. Notch signalling becomes transiently attenuated during long-term memory consolidation in adult Wistar rats. Neurobiol Learn Mem. 2007;88: 342–351. 10.1016/j.nlm.2007.04.006 17543552

[32] Dahlhaus M, Hermans JM, Van Woerden LH, Saiepour MH, Nakazawa K, Mansvelder HD, et al Notch1 signaling in pyramidal neurons regulates synaptic connectivity and experience-dependent modifications of acuity in the visual cortex. J Neurosci. 2008;28: 10794–10802. 10.1523/JNEUROSCI.1348-08.2008 18945887 PMC6671381

[33] Pavlopoulos E, Anezaki M, Skoulakis EMC. Neuralized is expressed in the alpha/beta lobes of adult Drosophila mushroom bodies and facilitates olfactory long-term memory formation. Proc Natl Acad Sci USA. 2008;105: 14674–14679. 10.1073/pnas.0801605105 18794519 PMC2567224

[34] Matsuno M, Horiuchi J, Tully T, Saitoe M. The Drosophila cell adhesion molecule klingon is required for long-term memory formation and is regulated by Notch. Proc Natl Acad Sci USA. 2009;106: 310–315. 10.1073/pnas.0807665106 19104051 PMC2606903

[35] Alberi L, Liu S, Wang Y, Badie R, Smith-Hicks C, Wu J, et al Activity-induced Notch signaling in neurons requires Arc/Arg3.1 and is essential for synaptic plasticity in hippocampal networks. Neuron. 2011;69: 437–444. 10.1016/j.neuron.2011.01.004 21315255 PMC3056341

[36] Yoon K-J, Lee H-R, Jo YS, An K, Jung S-Y, Jeong M-W, et al Mind bomb-1 is an essential modulator of long-term memory and synaptic plasticity via the Notch signaling pathway. Mol Brain. 2012;5: 40 10.1186/1756-6606-5-40 23111145 PMC3541076

[37] Sargin D, Botly LCP, Higgs G, Marsolais A, Frankland PW, Egan SE, et al Disrupting Jagged1–Notch signaling impairs spatial memory formation in adult mice. Neurobiol Learn Mem. 2013;103: 39–49. 10.1016/j.nlm.2013.03.001 23567106

[38] Zhang J, Little CJ, Tremmel DM, Yin JCP, Wesley CS. Notch-inducible hyperphosphorylated CREB and its ultradian oscillation in long-term memory formation. J Neurosci. 2013;33: 12825–12834. 10.1523/JNEUROSCI.0783-13.2013 23904617 PMC3728690

[39] Brai E, Marathe S, Zentilin L, Giacca M, Nimpf J, Kretz R, et al Notch1 activity in the olfactory bulb is odour-dependent and contributes to olfactory behaviour. Eur J Neurosci. 2014;40: 3436–3449. 10.1111/ejn.12719 25234246

[40] Dias BG, Goodman JV, Ahluwalia R, Easton AE, Andero R, Ressler KJ. Amygdala-Dependent Fear Memory Consolidation via miR-34a and Notch Signaling. Neuron. Elsevier Inc; 2014;: 1–13. 10.1016/j.neuron.2014.07.019 PMC4172484 25123309

[41] Liu S, Wang Y, Worley PF, Mattson MP, Gaiano N. The canonical Notch pathway effector RBP-J regulates neuronal plasticity and expression of GABA transporters in hippocampal networks. Hippocampus. 2015;25: 670–678. 10.1002/hipo.22402 25515406 PMC4412774

[42] Zhang J, Yin JCP, Wesley CS. Notch Intracellular Domain (NICD) Suppresses Long-Term Memory Formation in Adult Drosophila Flies. Cellular and Molecular Neurobiology. Springer US; 2015;: 1–6. 10.1007/s10571-015-0183-9 PMC4504794 25791355

[43] Kopan R, Ilagan MXG. The canonical Notch signaling pathway: unfolding the activation mechanism. Cell. 2009;137: 216–233. 10.1016/j.cell.2009.03.045 19379690 PMC2827930

[44] Hayward P, Brennan K, Sanders P, Balayo T, DasGupta R, Perrimon N, et al Notch modulates Wnt signalling by associating with Armadillo/beta-catenin and regulating its transcriptional activity. Development. 2005;132: 1819–1830. 10.1242/dev.01724 15772135 PMC2500123

[45] Le Gall M, De Mattei C, Giniger E. Molecular separation of two signaling pathways for the receptor, Notch. Dev Biol. 2008;313: 556–567. 10.1016/j.ydbio.2007.10.030 18062953 PMC2262048

[46] Perumalsamy LR, Nagala M, Banerjee P, Sarin A. A hierarchical cascade activated by non-canonical Notch signaling and the mTOR-Rictor complex regulates neglect-induced death in mammalian cells. Cell Death Differ. 2009;16: 879–889. 10.1038/cdd.2009.20 19265851

[47] Kuzina I, Song JK, Giniger E. How Notch establishes longitudinal axon connections between successive segments of the Drosophila CNS. Development. 2011;138: 1839–1849. 10.1242/dev.062471 21447553 PMC3074455

[48] Kwon C, Cheng P, King IN, Andersen P, Shenje L, Nigam V, et al Notch post-translationally regulates β-catenin protein in stem and progenitor cells. Nature Cell Biology. 2011;13: 1244–1251. 10.1038/ncb2313 21841793 PMC3187850

[49] Song JK, Giniger E. Noncanonical Notch function in motor axon guidance is mediated by Rac GTPase and the GEF1 domain of Trio. Dev Dyn. 2011;240: 324–332. 10.1002/dvdy.22525 21246649 PMC3070923

[50] Lee KS, Wu Z, Song Y, Mitra SS, Feroze AH, Cheshier SH, et al Roles of PINK1, mTORC2, and mitochondria in preserving brain tumor-forming stem cells in a noncanonical Notch signaling pathway. Genes Dev. 2013;27: 2642–2647. 10.1101/gad.225169.113 24352421 PMC3877754

[51] Lieber T, Kidd S, Struhl G. DSL-Notch signaling in the Drosophila brain in response to olfactory stimulation. Neuron. 2011;69: 468–481. 10.1016/j.neuron.2010.12.015 21315258 PMC3216490

[52] Kazama H, Wilson RI. Homeostatic matching and nonlinear amplification at identified central synapses. Neuron. 2008;58: 401–413. 10.1016/j.neuron.2008.02.030 18466750 PMC2429849

[53] Mejia M, Heghinian MD, Marí F, Godenschwege TA. New tools for targeted disruption of cholinergic synaptic transmission in Drosophila melanogaster. PLoS ONE. 2013;8: e64685 10.1371/journal.pone.0064685 23737994 PMC3667824

[54] Chen T-W, Wardill TJ, Sun Y, Pulver SR, Renninger SL, Baohan A, et al Ultrasensitive fluorescent proteins for imaging neuronal activity. Nature. 2013;499: 295–300. 10.1038/nature12354 23868258 PMC3777791

[55] Friggi-Grelin F, Coulom H, Meller M, Gomez D, Hirsh J, Birman S. Targeted gene expression in Drosophila dopaminergic cells using regulatory sequences from tyrosine hydroxylase. J Neurobiol. 2003;54: 618–627. 10.1002/neu.10185 12555273

[56] McNabb SL, Truman JW. Light and peptidergic eclosion hormone neurons stimulate a rapid eclosion response that masks circadian emergence in Drosophila. J Exp Biol. 2008;211: 2263–2274. 10.1242/jeb.015818 18587121 PMC2760273

[57] Overstreet E. Fat facets and Liquid facets promote Delta endocytosis and Delta signaling in the signaling cells. Development. 2004;131: 5355–5366. 10.1242/dev.01434 15469967

[58] Wang W, Struhl G. Drosophila Epsin mediates a select endocytic pathway that DSL ligands must enter to activate Notch. Development. 2004;131: 5367–5380. 10.1242/dev.01413 15469974

[59] Couto A, Alenius M, Dickson BJ. Molecular, Anatomical, and Functional Organization of the Drosophila Olfactory System. Current Biology. 2005;15: 1535–1547. 10.1016/j.cub.2005.07.034 16139208

[60] Lieber T, Kidd S, Alcamo E, Corbin V, Young MW. Antineurogenic phenotypes induced by truncated Notch proteins indicate a role in signal transduction and may point to a novel function for Notch in nuclei. Genes Dev. 1993;7: 1949–1965. 8406001 10.1101/gad.7.10.1949

[61] Kidd S, Lieber T, Young MW. Ligand-induced cleavage and regulation of nuclear entry of Notch in Drosophila melanogaster embryos. Genes Dev. 1998;12: 3728–3740. 9851979 10.1101/gad.12.23.3728 PMC317253

[62] Morsut L, Roybal KT, Xiong X, Gordley RM, Coyle SM, Thomson M, et al Engineering Customized Cell Sensing and Response Behaviors Using Synthetic Notch Receptors. Cell. Elsevier Inc; 2016;: 1–19. 10.1016/j.cell.2016.01.012 PMC4752866 26830878

[63] Iwai Y, Usui T, Hirano S, Steward R, Takeichi M, Uemura T. Axon patterning requires DN-cadherin, a novel neuronal adhesion receptor, in the Drosophila embryonic CNS. Neuron. 1997;19: 77–89. 9247265 10.1016/s0896-6273(00)80349-9

[64] Potter CJ, Tasic B, Russler EV, Liang L, Luo L. The Q system: a repressible binary system for transgene expression, lineage tracing, and mosaic analysis. Cell. 2010;141: 536–548. 10.1016/j.cell.2010.02.025 20434990 PMC2883883

[65] Tan Z-J, Peng Y, Song H-L, Zheng J-J, Yu X. N-cadherin-dependent neuron-neuron interaction is required for the maintenance of activity-induced dendrite growth. Proceedings of the National Academy of Sciences. 2010;107: 9873–9878. 10.1073/pnas.1003480107 PMC2906874 20457910

[66] Yew JY, Wang Y, Barteneva N, Dikler S, Kutz-Naber KK, Li L, et al Analysis of neuropeptide expression and localization in adult drosophila melanogaster central nervous system by affinity cell-capture mass spectrometry. J Proteome Res. 2009;8: 1271–1284. 10.1021/pr800601x 19199706 PMC2693453

[67] Carlsson MA, Diesner M, Schachtner J, Nässel DR. Multiple neuropeptides in the Drosophila antennal lobe suggest complex modulatory circuits. J Comp Neurol. 2010;518: 3359–3380. 10.1002/cne.22405 20575072

[68] Nässel DR, Winther AME. Drosophila neuropeptides in regulation of physiology and behavior. Prog Neurobiol. 2010;92: 42–104. 10.1016/j.pneurobio.2010.04.010 20447440

[69] Das S, Sadanandappa MK, Dervan A, Larkin A, Lee JA, Sudhakaran IP, et al Plasticity of local GABAergic interneurons drives olfactory habituation. Proc Natl Acad Sci USA. 2011;108: E646–E654. 10.1073/pnas.1106411108/-/DCSupplemental 21795607 PMC3169145

[70] Davis GW. Homeostatic control of neural activity: from phenomenology to molecular design. Annu Rev Neurosci. 2006;29: 307–323. 10.1146/annurev.neuro.28.061604.135751 16776588

[71] del Álamo D, Rouault H, Schweisguth F. Mechanism and significance of cis-inhibition in Notch signalling. Curr Biol. 2011;21: R40–7. 10.1016/j.cub.2010.10.034 21215938

[72] Ni J-Q, Zhou R, Czech B, Liu L-P, Holderbaum L, Yang-Zhou D, et al A genome-scale shRNA resource for transgenic RNAi in Drosophila. Nat Meth. Nature Publishing Group; 2011;8: 405–407. 10.1038/nmeth.1592 PMC3489273 21460824

[73] Pfeiffer BD, Ngo T-TB, Hibbard KL, Murphy C, Jenett A, Truman JW, et al Refinement of tools for targeted gene expression in Drosophila. Genetics. 2010;186: 735–755. 10.1534/genetics.110.119917 20697123 PMC2942869

[74] Fishilevich E, Vosshall LB. Genetic and functional subdivision of the Drosophila antennal lobe. Curr Biol. 2005;15: 1548–1553. 10.1016/j.cub.2005.07.066 16139209

[75] Lee T, Luo L. Mosaic analysis with a repressible cell marker for studies of gene function in neuronal morphogenesis. Neuron. 1999;22: 451–461. 10197526 10.1016/s0896-6273(00)80701-1

[76] Marin EC. Developmentally programmed remodeling of the Drosophila olfactory circuit. Development. 2005;132: 725–737. 10.1242/dev.01614 15659487

[77] Sweeney ST, Broadie K, Keane J, Niemann H, O'Kane CJ. Targeted expression of tetanus toxin light chain in Drosophila specifically eliminates synaptic transmission and causes behavioral defects. Neuron. 1995;14: 341–351. 7857643 10.1016/0896-6273(95)90290-2

[78] McGuire SE, Le PT, Osborn AJ, Matsumoto K, Davis RL. Spatiotemporal rescue of memory dysfunction in Drosophila. Science. 2003;302: 1765–1768. 10.1126/science.1089035 14657498

[79] Dietzl G, Chen D, Schnorrer F, Su K-C, Barinova Y, Fellner M, et al A genome-wide transgenic RNAi library for conditional gene inactivation in Drosophila. Nature. 2007;448: 151–156. 10.1038/nature05954 17625558

[80] Motulsky HJ, Brown RE. Detecting outliers when fitting data with nonlinear regression—a new method based on robust nonlinear regression and the false discovery rate. BMC Bioinformatics. 2006;7: 123 10.1186/1471-2105-7-123 16526949 PMC1472692

[81] Wagh DA, Rasse TM, Asan E, Hofbauer A, Schwenkert I, Dürrbeck H, et al Bruchpilot, a protein with homology to ELKS/CAST, is required for structural integrity and function of synaptic active zones in Drosophila. Neuron. 2006;49: 833–844. 10.1016/j.neuron.2006.02.008 16543132

[82] Xie X, Cho B, Fischer JA. Drosophila Epsin's role in Notch ligand cells requires three Epsin protein functions: The lipid binding function of the ENTH domain, a single Ubiquitin interaction motif, and a subset of the C-terminal protein binding modules. Dev Biol. Elsevier Inc; 2012;363: 399–412. 10.1016/j.ydbio.2012.01.004 PMC3288543 22265678

