# Supplementary figures and images for: Mechanism of Notch Pathway Activation and Its Role in the Regulation of Olfactory Plasticity in Drosophila melanogaster
Source: PLoS One. 2016 Mar 17;11(3):e0151279. doi: 10.1371/journal.pone.0151279 (PMC4795742; doi:10.1371/journal.pone.0151279)

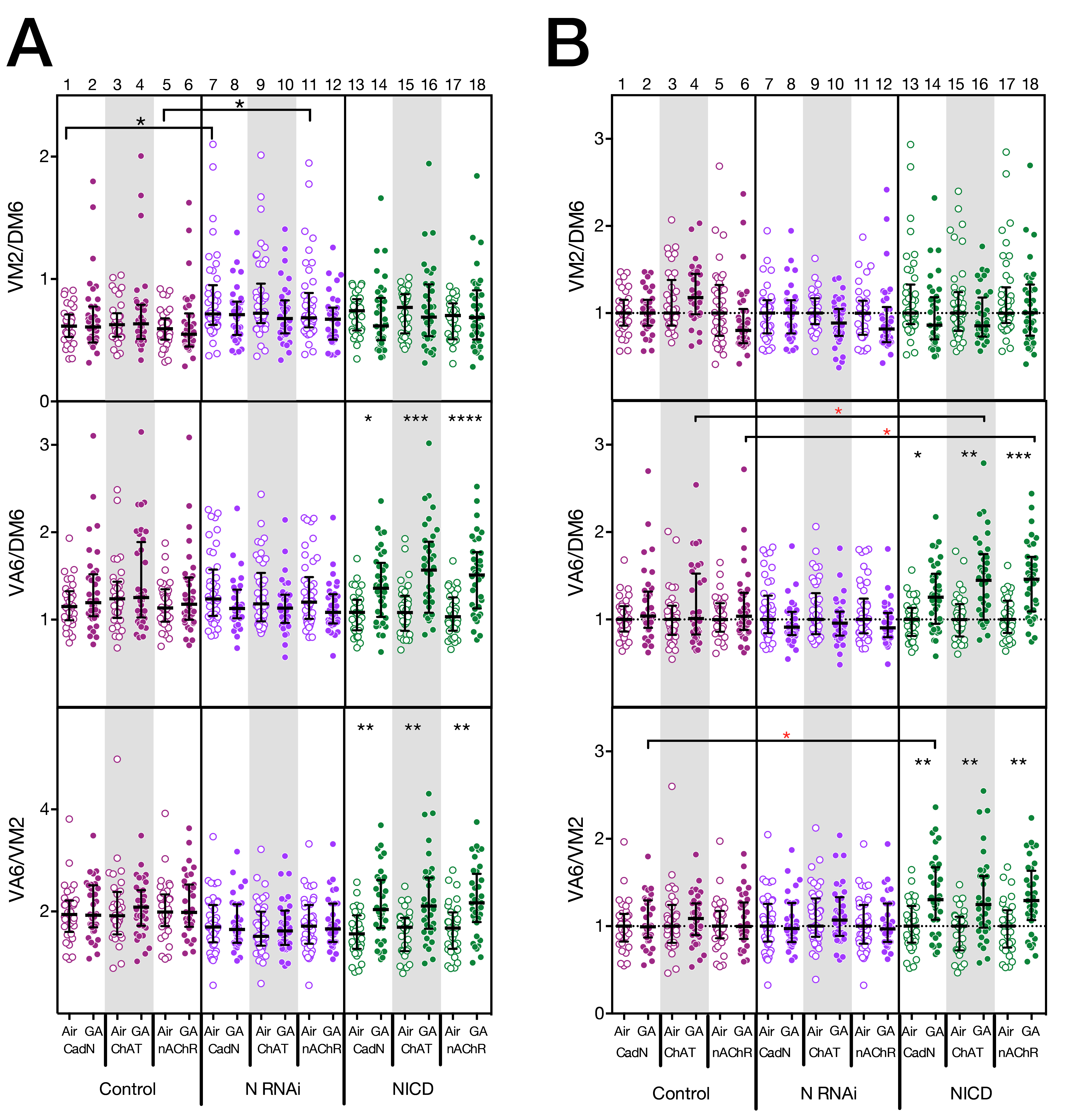

Supplement: S1 Fig — Female flies expressing N RNAi or NICD in VA6 ORNs, or control flies, were exposed to 1% GA in paraffin oil or paraffin oil alone for four days. Brains were then co-reacted with anti-cadN, anti-ChAT and A488-BTX to visualize nAChRs. (A) For each brain we quantified the pixel intensities of VA6, VM2 and DM6 and then determined the ratios of all three combinations. The ratios are presented as scatter plots. (B) Each ratio for GA and air exposed flies was normalized to the median value of the air exposed flies. The ratios are presented as scatter plots. Normalized air exposed flies by definition have a median of one, which is indicated by the dashed line. Statistical significance was determined by the Kruskal-Wallis test with Dunn’s correction for multiple comparisons. Controls are in magenta, N RNAi are purple and NICD are green. Open circles are air exposed and filled circles are geranyl acetate exposed flies. Flies were Or82a-GAL4 with either UAS.Val20, UAS.N shRNA or UAS.NICD. (TIF) [file pone.0151279.s001.tif]
